# A cytokine protein-protein interaction network for identifying key molecules in rheumatoid arthritis

**DOI:** 10.1371/journal.pone.0199530

**Published:** 2018-06-21

**Authors:** Venugopal Panga, Srivatsan Raghunathan

**Affiliations:** 1 Institute of Bioinformatics and Applied Biotechnology (IBAB), Biotech Park, Electronics City Phase I, Bengaluru, Karnataka, India; 2 Manipal Academy of Higher Education, Manipal, Karnataka, India; Macau University of Science and Technology, MACAO

## Abstract

Rheumatoid arthritis (RA) is a chronic inflammatory disease of the synovial joints. Though the current RA therapeutics such as disease-modifying antirheumatic drugs (DMARDs), nonsteroidal anti-inflammatory drugs (NSAIDs) and biologics can halt the progression of the disease, none of these would either dramatically reduce or cure RA. So, the identification of potential therapeutic targets and new therapies for RA are active areas of research. Several studies have discovered the involvement of cytokines in the pathogenesis of this disease. These cytokines induce signal transduction pathways in RA synovial fibroblasts (RASF). These pathways share many signal transducers and their interacting proteins, resulting in the formation of a signaling network. In order to understand the involvement of this network in RA pathogenesis, it is essential to identify the key transducers and their interacting proteins that are part of this network. In this study, based on a detailed literature survey, we have identified a list of 12 cytokines that induce signal transduction pathways in RASF. For these cytokines, we have built a signaling network using the protein-protein interaction (PPI) data that was obtained from public repositories such as HPRD, BioGRID, MINT, IntAct and STRING. By combining the network centrality measures with the gene expression data from the RA related microarrays that are available in the open source Gene Expression Omnibus (GEO) database, we have identified 24 key proteins of this signaling network. Two of these 24 are already drug targets for RA, and of the remaining, 12 have direct PPI links to some of the current drug targets of RA. Therefore, these key proteins seem to be crucial in the pathogenesis of RA and hence might be treated as potential drug targets.

## Introduction

RA is a debilitating chronic inflammatory synovial joint disease that affects about 1% of the world’s population [1]. The disease usually affects the small joints of the hands and feet. The etiology of the disease is unknown. The chronic inflammation causes invasion of synovial membrane toward articular bone which results in the formation of a layer of granulation tissue, called pannus. Further, the inflammation would induce irreversible damage of the synovium, which leads to dysfunction of the joints [1–2]. The current RA therapeutics can suppress inflammation and the damage of cartilage and bone but cannot cure the disease. Further, hepatotoxicity, cardiotoxicity and gastrointestinal effects are the clinical side effects of some of these treatments [3–5]. The lack of a clear understanding of the pathogenesis of the disease remains an obstacle for discovering effective treatments for RA.

Along with RASF, various immune cells, such as B cells, T cells, mast cells, macrophages, dendritic cells and natural killer (NK) cells are activated in RA [6]. Many of these cells produce cytokines, which are involved in the pathogenesis of the disease. Some of these cytokines are pro-inflammatory while others are anti-inflammatory. In RA, cytokines induce autoimmunity, chronic inflammation and eventual joint damage. Many cytokines such as tumor necrosis factor (TNF), interferon gamma (IFNγ), several interleukins—IL-1β, IL-4, IL-6, IL-7, IL-12, IL-13, IL-15, IL-18, IL-23 and transforming growth factor beta (TGFβ) are expressed in the synovial tissues [1]. Also, the cytokine levels in the synovial tissues are altered at various stages of the disease. For instance, in early RA the levels of IL-13 and IL-4 are elevated, whereas in the later stages they are present at low levels [7]. Some of these cytokines act in a synergistic fashion to augment inflammation. For instance, IL-17, IL-1β and TNF synergistically activate synovial fibroblasts leading to the production of inflammatory mediators [8]. Several of these cytokines induce signal transduction pathways in RASF, which lead to the activation of their respective transcription factors. The activated transcription factors induce the expression of the genes that encode inflammatory mediators such as C-X-C motif ligands—CXCL8, CXCL9, CXCL10, CXCL11, CXCL12 and C-C motif ligand 5 (CCL5)—and articular cartilage degrading enzymes such as matrix metalloproteinases MMP1 and MMP3 [9–33].

RA is a complex phenotype that can be caused by a combination of genetic and environmental stresses on complex biological networks [34–35]. The pro- and anti-inflammatory cytokines form one such complex network. The cytokines interact with one another via their signal transduction pathways. Many signal transducers are shared by these pathways, thus forming a network. However, which of these signal transducers can be targeted for the effective treatment of RA has not been fully established. Building the cytokine network and identifying the key molecules in it would be a major step toward the holistic understanding of the cytokine pathways in RA. The cytokine network can be built using protein-protein interaction (PPI) data that is available in the public repositories such as the human protein reference database (HPRD), the biological general repository for interaction datasets (BioGRID), the molecular interaction (MINT), IntAct, STRING and a tissue-specific human protein interaction dataset called CRG [36–41]. By combining network analysis methods and the RA gene expression profiling data that is present in the GEO database, we can come up with strategies for identifying certain key molecules that are part of the cytokine signaling network. In earlier studies, some efforts have been made to identify key molecules in complex diseases like tuberculosis and cancer using network analysis methods [42–43]. However, such studies are limited in RA. In order to fill this gap, we had earlier created a cytokine signaling network based on literature curation and evaluated the activity of the cytokine signaling pathways using gene expression profiling data [44]. Further, Hwang et al. have emphasized the use of existing gene expression data for pathway evaluation in RA [45].

In this study, using publicly available PPI data, we have built a signaling network associated with the cytokines and their transcription factors that are active in RASF. Using network centrality measures, we have identified some important molecules of this network. Further, the activity of these molecules was assessed using the RASF gene expression profiling data that is available in GEO. Finally, the proteins with higher centrality scores and with differential gene expression were considered the key molecules.

## Methods

### Creation of a human PPI database

We created a human PPI database by extracting the interactions from six publicly available databases, namely HPRD, BioGRID, IntAct, MINT, STRING and CRG [36–41]. From each database, only the experimentally determined physical interactions in human cells were considered. The experimental methods used for determining the protein interactions that are listed in each database are given in the S1 File. All these interactions were merged into a single database by converting the protein identifiers of individual databases into gene symbols. In HPRD and BioGRID the proteins are represented with their respective gene symbols. In other databases the different protein identifiers were converted into gene symbols before merging. For instance, in IntAct and MINT databases, the proteins are identified with their respective UniProtKB entries. They were converted into gene symbols by using a HUGO gene nomenclature committee (HGNC) custom downloaded file containing gene symbols and UniProtKB entries [46]. Similarly, in the STRING and CRG databases the proteins are represented with the Ensembl protein and Ensembl gene identifiers respectively, and were converted into gene symbols with the aid of the Ensembl Biomart project [47]. All these interactions have been merged based on their gene symbols to create a final human PPI database. The database is in the S2 File.

### Creation of a synovial tissue-specific PPI database for the plasma membrane and cytoplasm

In this study, we have focused on creating a cytokine signaling network starting from the binding of the cytokines to their cell surface receptors and ending with the activation of the transcription factors in the cytoplasm. Therefore we aim to create a PPI interactome that is specific to the plasma membrane and the cytoplasm. For this, only the interactions of those proteins that get localized to the cytoplasm and the plasma membrane were extracted from the human PPI database using the subcellular localization data present in the mammalian protein subcellular localization database, LOCATE [48]. We also extracted the interactions of many cytokine receptors and transcription factors that are not listed in LOCATE. A list of the genes of this interactome, called the ‘G-list’, was prepared. Furthermore, to make this interactome specific to the synovial tissue, we computed the co-expression of the interacting partners of these interactions by analyzing the RA related microarray gene expression data obtained from GEO. All the microarray datasets chosen in this study are based on the Affymetrix platform. Further details about the microarray datasets and their analysis are provided at the end of the ‘Methods’ section. For every microarray dataset, we did the following; for a pair of genes in the G-list we computed the Pearson correlation coefficient of the gene expression values across all RA disease samples. The correlations were computed for all possible pairs in the G-list. This was repeated for all the datasets. Only those pairs that had experimentally determined PPI interactions and with a Pearson correlation coefficient > 0.7 in at least one microarray dataset were considered as co-expressed in the synovial tissues. Finally, a synovial tissue-specific PPI database was created by selecting the interactions of the co-expressed interacting proteins.

### Creation of cytokine PPI network (CPPIN) in RASF

Using the created synovial tissue-specific PPI database, we have built a network with proteins as nodes and their interactions as edges. The open source ‘igraph’ package in ‘R’ and python scripts were used for this purpose. We named this network the ‘synovial PPI network’ (SPPIN).

Based on a literature search in PubMed, we identified 12 active cytokines and 8 of their transcription factors in RASF. Details of these cytokines and their transcription factors are described in the Results section and the list of search terms used is in S3 File.

Considering these cytokines and their transcription factors as sources and targets respectively, we extracted all the shortest paths from the sources to the targets from the SPPIN network using the Breadth-first search (BFS) algorithm. In addition to BFS, there are several other approaches for finding shortest paths in graphs. Some of them are Depth-first search (DFS), Dijkstra and A* [49]. DFS puts the visited vertices in a stack while BFS puts them in a queue. The BFS algorithm is generally used for finding shortest paths in unweighted PPI networks. In SPPIN, the sources and the targets are closer to one another, which is an ideal scenario for using the BFS algorithm. This algorithm is computationally faster for searching shortest paths in SPPIN.

**Fig 1:**
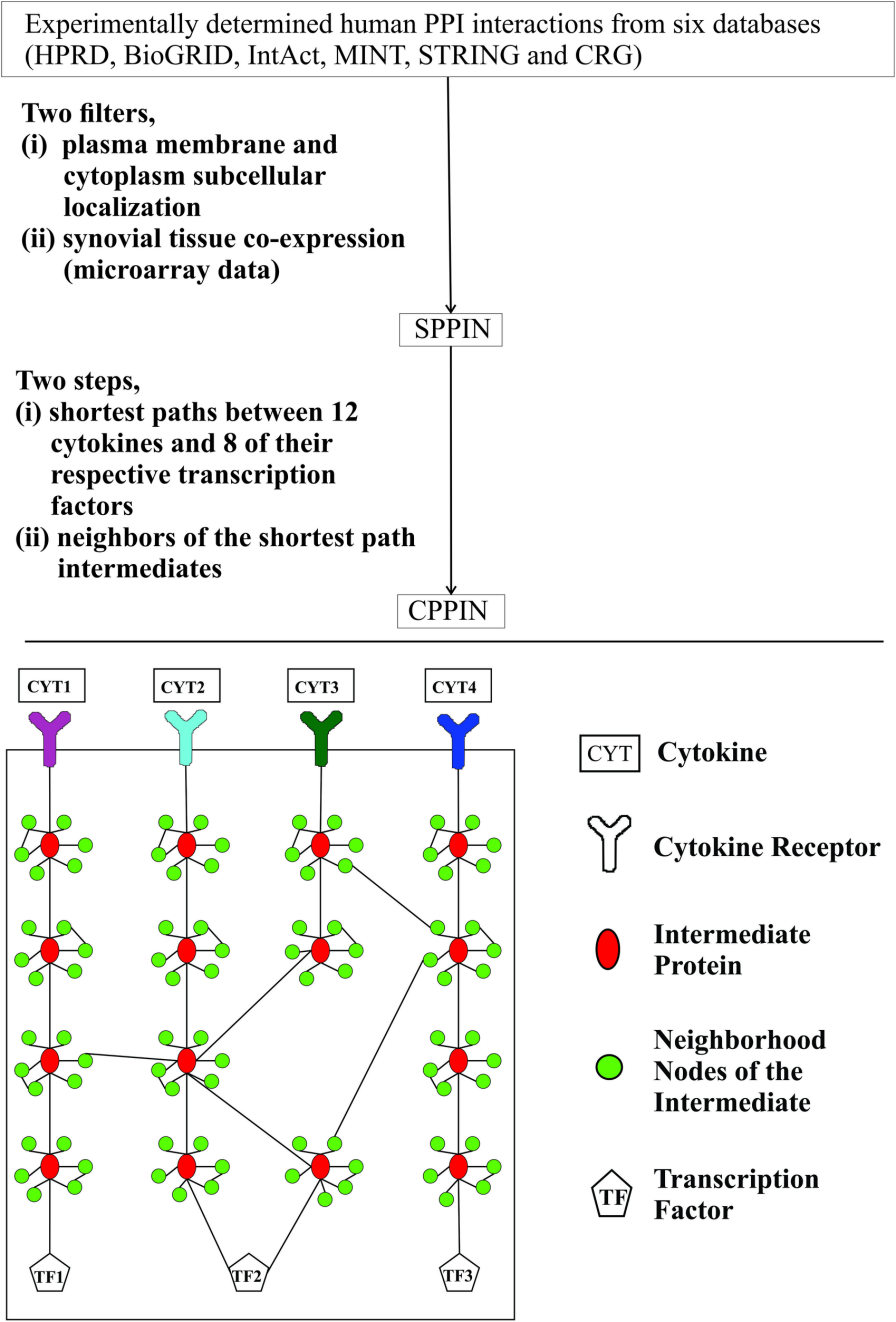
Building cytokine PPI network (CPPIN) in RA synovial fibroblasts (RASF). CPPIN was built using the publicly available PPI data. For building this network, 12 cytokines and eight of their target transcription factors that are active in RASF were considered. In the figure, the upper half shows the steps involved in the creation of CPPIN while the lower half is a representation of CPPIN. First, the network SPPIN was created by applying two filters to the collated interactions from the six PPI databases. The two filters are (i) plasma membrane and cytoplasm subcellular localization and (ii) synovial tissue co-expression using microarray data. For creating CPPIN from SPPIN, the shortest paths between cytokines and target transcription factors and the neighboring nodes of the shortest path intermediates were used. In the lower half of the figure, the rectangles represent cytokines, the Y-shaped symbols represent the cytokine receptors, the red colored vertical ovals represent the intermediate proteins that connect the cytokine receptors and the transcription factors, the green circles represent the neighborhood nodes of the intermediate proteins and the pentagons represent the target transcription factors of the cytokines.

Each shortest path extracted from SPPIN contains the receptor of the source cytokine, the target transcription factor and the intermediate proteins that connect them. If a cytokine receptor is encoded by more than one gene, all the shortest paths between each of the cytokine receptor genes and their respective transcription factors were considered. Similarly, if a transcription factor is encoded by more than one gene, all the shortest paths between the cytokine receptor and each of these genes were considered. There were a total of 103 distinct intermediate proteins in the shortest paths. Each of these intermediates was treated as a focal node and all of the latter’s immediate neighborhood nodes were isolated. Finally, we formed the cytokine network by connecting the cytokines, their transcription factors, the intermediates that connect them and the neighborhood nodes of the intermediates [Fig 1]. We named this network the ‘cytokine PPI network’ (CPPIN).

### Centrality measures

In order to identify the highly connected and central proteins in the network, we measured four important centralities of every node present in the network as described below.

#### Degree centrality

Degree centrality is the number of edges through which a node connects to other nodes within a network. Proteins with a high degree are connected to a large number of other proteins. In PPI networks, proteins with a higher degree are considered ‘essential proteins’ or ‘hubs’ as they are located at the center of the network [50]. Considering the pair-wise interactions of ‘n’ nodes, the degree centrality of a node p_k_ is calculated using the following equation [51].

$$CD(p_{k})=\frac{\sum_{i=1}^{n}a(p_{i},p_{k})}{n-1}$$ (1)

Where a(pi, pk) = 1 if and only if protein pi and protein pk are connected by an edge. Otherwise it is 0. ‘n’ is the total number of nodes present in the network.

#### Betweenness centrality

Betweenness centrality is the measure of the number of shortest paths that pass through a node within a network. Nodes with high betweenness, called ‘bottlenecks’, control the flow of information within a network [52]. The betweenness of a node ‘n’ takes the node pairs such as (n1, n2) and calculates all the shortest paths that go through ‘n’ to connect n1 and n2. The betweenness of a node ‘n’ is calculated using the following equation.

$$CB(n)=\sum_{n1\neq n\neq n2}\frac{{}_{{}_{g_{n1,n,n2}}}}{g_{n1,n2}}$$ (2)

Where, ‘g_n1, n, n2_’ is the number of shortest paths that pass through node ‘n’ and ‘g_n1, n2_’ is the total number of shortest paths.

#### Closeness centrality

Closeness centrality is a measure of the average distance of all the shortest paths between a node and every other node within a network. It gives how far a certain node is from all other nodes [51]. This is calculated using the following equation.

$$CC(n)=\frac{1}{\sum_{i\neq n}g_{n}i}$$ (3)

Where, g_n_i represents the shortest paths between the node ‘n’ and every other node i. Closeness centralities are measured for all the nodes present within the CPPIN network.

#### Eigenvector centrality

Eigenvector centrality measures the influence of a node within a network. The node with a high eigenvector is considered the central and influential node as it is connected to many other central nodes [53]. Eigenvector centrality scores are the elements of the first eigenvector of the adjacency matrix of a network.

### Microarray data analysis

In this study, we have considered five Affymetrix RA related microarray datasets that are available in the NCBI GEO database. They are GSE7307, GSE55457, GSE55235, GSE12021 (HGU133A) and GSE12021 (HGU133B) (Table 1). These microarray experiments were carried out on RA and normal synovial fibroblasts by other workers. The RA samples used in these studies were obtained by tissue excision upon joint replacement/synovectomy surgery from RA patients whereas the control samples were obtained from either postmortem joints or traumatic joint injury cases. We re-analyzed these datasets using the R/Bioconductor statistical package. All the datasets were normalized using two algorithms, MAS5 and RMA, separately. The differential expression of the genes between RA and control groups was computed using the two sample independent t-test.

**Table 1:** Details of microarray datasets used in this study.

S.No.	GEO Accession	PubMed ID	Platform	Probe Number	Number of Samples	
					RA	Control
1	GSE7307	-	Affymetrix Human Genome U133 Plus 2.0 Array	54675	5	9
2	GSE12021	18721452	Affymetrix Human Genome U133A Array	22283	12	9
3	GSE12021	18721452	Affymetrix Human Genome U133B Array	22645	12	4
4	GSE55457	24690414	Affymetrix Human Genome U133A Array	22283	13	10
5	GSE55235	24690414	Affymetrix Human Genome U133A Array	22283	10	10

For the co-expression analysis described above, we considered the RMA normalized expression values from the disease samples of each microarray dataset.

A gene is said to be differentially expressed in a dataset if it satisfies the following criteria: (i) Gene should have a P-value < 0.05 and a fold-change > 1.5 for up or down regulation. (ii) A gene is said to be up-regulated if it shows up-regulation by both the normalization methods or up-regulation in one and below the fold-change threshold in the other. (iii) Similarly, a gene is said to be down-regulated if it shows down-regulation by both the normalization methods or down-regulation in one and below the fold-change threshold in the other.

The same criteria were applied across the datasets to decide whether a gene is up/down regulated in each one of them.

## Results

### Construction of the CPPIN network

As explained in the ‘Methods’, we combined interactions from all the six resources to obtain a complete dataset. By doing this, we obtained 77218 interactions from CRG, 39042 from HPRD, 298802 from BioGRID, 89355 from STRING and 45896 from both the IntAct and MINT databases. Overall, we considered 363,476 non-redundant interactions from the six resources (S2 File). Interactions from all these six resources seem to be comprehensive. Then, two filters were applied to this data: (i) plasma membrane and cytoplasm subcellular localization and (ii) synovial tissue co-expression. This resulted in a synovial tissue-specific interactome with 7939 interactions. Using this, we built the synovial tissue-specific PPI network, SPPIN. In this network, the proteins and their interactions are represented as nodes and edges respectively.

Based on a literature survey, we have identified 12 cytokines and eight of their target transcription factors that are active in RASF (Table 2). These cytokines stimulate the RASF and activate their respective transcription factors. We considered these cytokines as the sources and their transcription factors as the targets in SPPIN. We then extracted a total of 139 shortest paths that pass through the cytokine receptors between these sources and targets. The number of intermediates on all the shortest paths was 103. The number of times each of these intermediate proteins occurred was also determined (S4 File).

**Table 2:** Cytokines, their receptors, their transcription factors and shortest paths. All the combinations of the shortest paths from each of the cytokine receptors to their target transcription factor are considered.

S.No.	Cytokine	Cytokine Receptor(s)	Transcription Factor	Subunits/forms of the transcription factor	No. of shortest paths	Reference(s)
1	TNF	TNFRSF1A, TNFRSF1B	NF-κB	NFKB1, NFKB2, RELA, RELB, REL	10	[9–12, 54–66]
2	IL-18	IL18R1, IL18RAP	NF-κB	NFKB1, NFKB2, RELA, RELB, REL	10	[13]
3	IL-17	IL17RA, IL17RC	NF-κB	NFKB1, NFKB2, RELA, RELB, REL	10	[11, 14–15, 67–68]
4	IL-27	IL27RA, IL6ST	STAT1	STAT1	2	[16]
5	IL-1α/β	IL1R1	NF-κB	NFKB1, NFKB2, RELA, RELB, REL	5	[11–12, 17–18, 58, 62, 69–75]
6	TNF	TNFRSF1A, TNFRSF1B	AP-1	JUN, JUNB, FOS, FOSB, FOSL1, FOSL2	12	[11, 16, 17, 54]
7	IL-1β	IL1R1	AP-1	JUN, JUNB, FOS, FOSB, FOSL1, FOSL2	6	[11, 16, 18–20, 71–72, 74]
8	IL-17	IL17RA, IL17RC	AP-1	JUN, JUNB, FOS, FOSB, FOSL1, FOSL2	12	[11, 15]
9	TNF	TNFRSF1A, TNFRSF1B	IRF3	IRF3	2	[19]
10	LIGHT (TNFSF14)	TNFRSF14	NF-κB	NFKB1, NFKB2, RELA, RELB, REL	5	[21–22]
11	IL-33	IL1RL1	NF-κB	NFKB1, NFKB2, RELA, RELB, REL	5	[23]
12	IL-17	IL17RA, IL17RC	STAT3	STAT3	2	[24, 76]
13	IL-1β	IL1R1	STAT1	STAT1	1	[16, 26]
14	IFNα/β	IFNAR1, IFNAR2	STAT1	STAT1	2	[10, 26–27]
15	IFNγ	IFNGR1, IFNGR2	STAT1	STAT1	2	[10, 26]
16	TNF	TNFRSF1A, TNFRSF1B	STAT1	STAT1	2	[16, 26]
17	IL-1α/β	IL1R1	IRF3	IRF3	1	[28]
18	IL-1α/β	IL1R1	IRF7	IRF7	1	[28]
19	IFNα/β	IFNAR1, IFNAR2	IRF3	IRF3	2	[28]
20	IFNα/β	IFNAR1, IFNAR2	IRF7	IRF7	2	[28]
21	TNF	TNFRSF1A, TNFRSF1B	IRF1	IRF1	2	[12]
22	IL-1β	IL1R1	IRF1	IRF1	1	[12]
23	TGF-β1	TGFBR1, TGFBR2	NF-κB	NFKB1, NFKB2, RELA, RELB, REL	10	[29–30]
24	TGF-β1	TGFBR1, TGFBR2	AP-1	JUN, JUNB, FOS, FOSB, FOSL1, FOSL2	12	[29]
25	TGF-β1	TGFBR1, TGFBR2	SMAD	SMAD2, SMAD3	4	[31, 77]
26	IL-21	IL2RG	STAT3	STAT3	1	[32, 78]
27	IL6	IL6R	STAT3	STAT3	1	[79]
28	TNF	TNFRSF1A, TNFRSF1B	STAT3	STAT3	2	[54]
29	IL-27	IL27RA, IL6ST	AP-1	JUN, JUNB, FOS, FOSB, FOSL1, FOSL2	12	[16]
					Total = 139	

TNFRSF1A and TNFRSF1B, tumor necrosis factor receptor superfamily member 1A and 1B; IL18R1, interleukin-18 receptor 1; IL18RAP, interleukin-18 receptor accessory protein; IL17RA, interleukin-17 receptor A; IL17RC, interleukin-17 receptor C; IL27RA, interleukin-27 receptor alpha; IL6ST, interleukin 6 signal transducer (glycoprotein 130); IL1R1, interleukin 1 receptor, type 1; TNFRSF14, tumor necrosis factor receptor superfamily member 14; IL1RL1, interleukin-1 receptor-like 1; IFNAR1, interferon-alpha receptor alpha chain; IFNAR2, interferon-alpha receptor beta chain; IFNGR1, interferon gamma receptor 1; IFNGR2, interferon gamma receptor 2; TGFBR1, TGF beta receptor 1; TGFBR2, TGF beta receptor 2; IL2RG, interleukin-2 receptor subunit gamma; IL6R, inteleukin-6 receptor; NF-κB, nuclear factor kappa-light-chain-enhancer of activated B cells; STAT1, signal transducer and activator of transcription 1; AP-1, activator protein 1; IRF3, interferon regulatory factor 3; STAT3, signal transducer and activator of transcription 3; IRF7, interferon regulatory factor 7; IRF1, interferon regulatory factor 1

We have also extracted the neighborhood nodes of the intermediates from the SPPIN network. The intermediates are considered seed nodes. All the nodes to which a seed node has a direct connection (path length 1) are extracted. Finally, we formed CPPIN by connecting the cytokines, their transcription factors, the 103 intermediates and the neighborhood nodes of the intermediates. This gave a network comprising 1204 nodes and 2155 edges. The edge list of this network is provided in S5 File.

### Central nodes of the CPPIN network and their activity in the RA synovium

**Fig 2:**
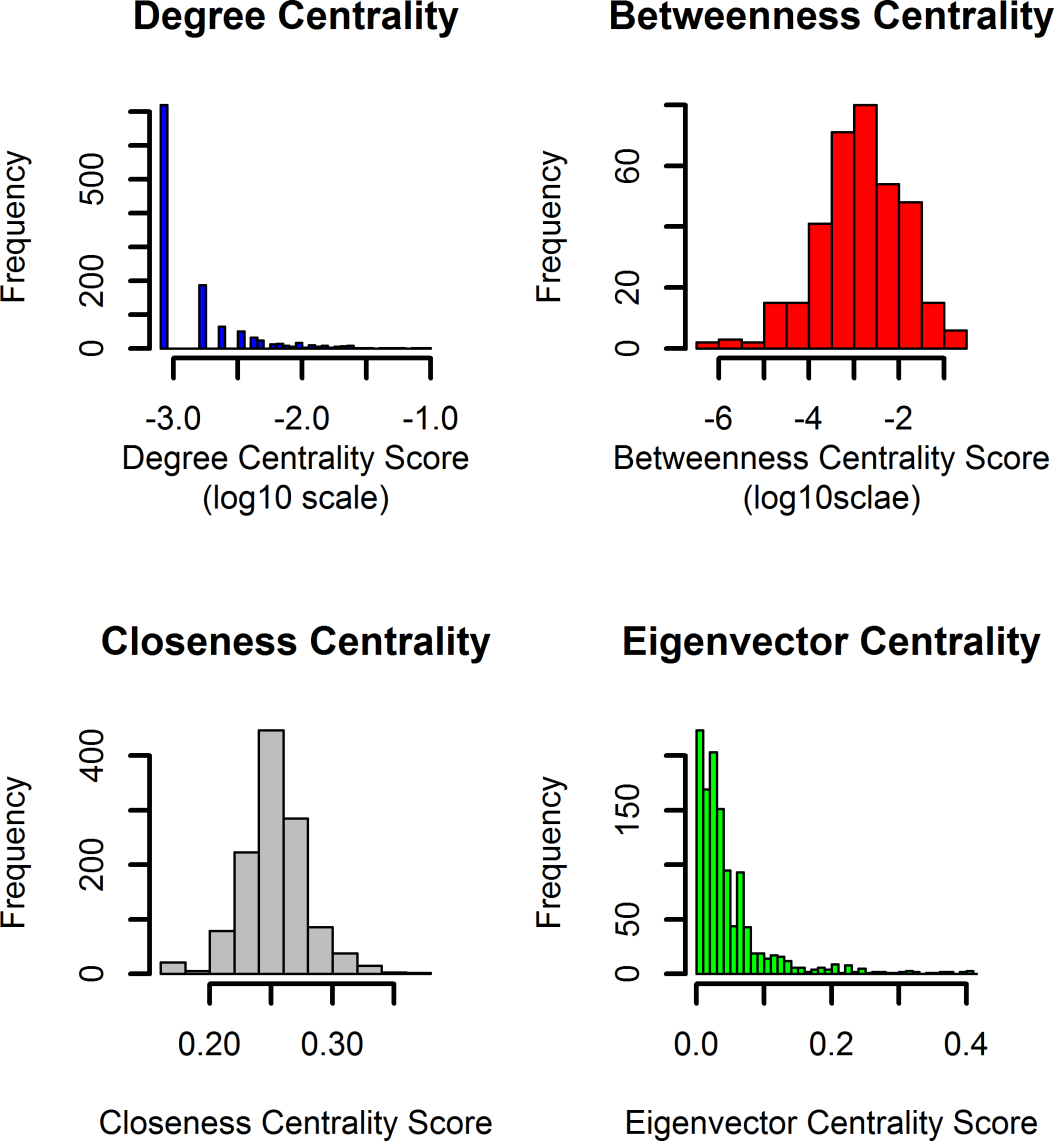
The histograms of the four centrality measures, degree, betweenness, closeness and eigenvector. The distributions of the four centrality measures are plotted as histograms. Taking the histogram as a reference, approximately 20% of the proteins with high centrality scores (toward the right side of the histogram) are extracted for differential expression analysis.

**Fig 3:**
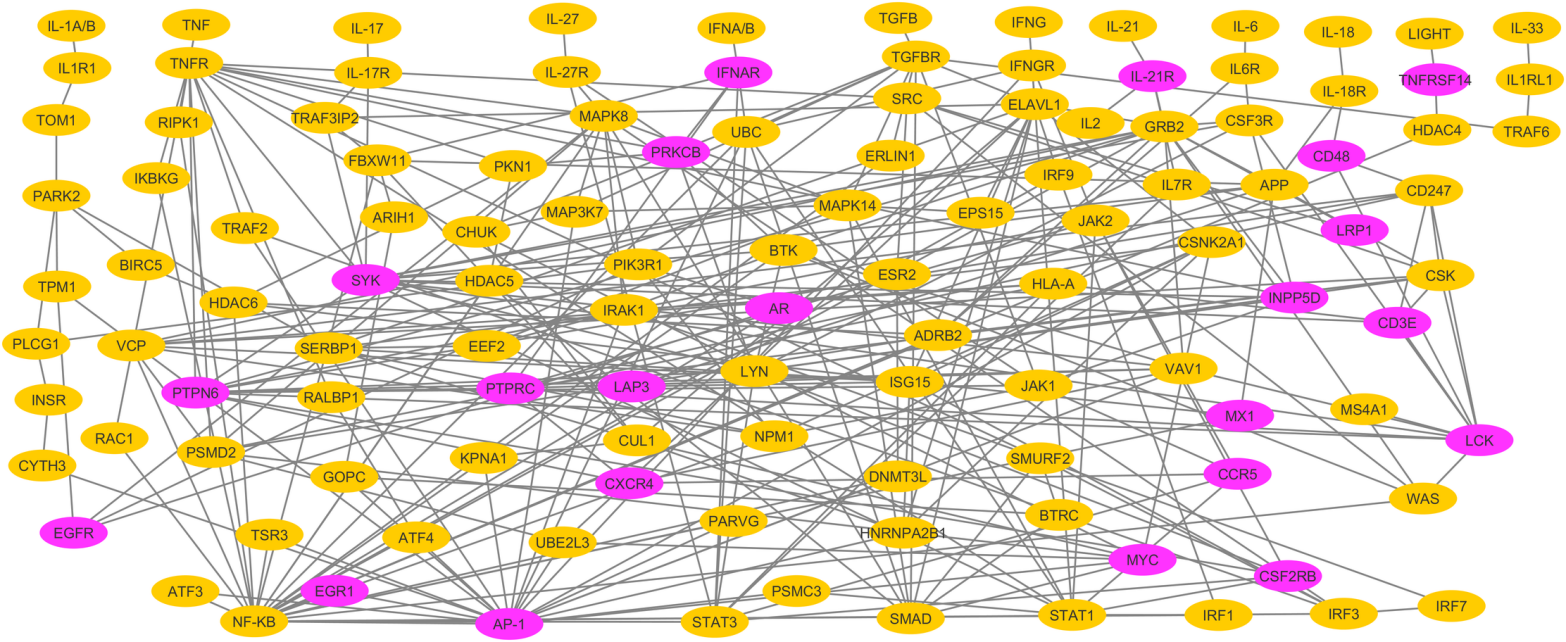
A concise interaction map for the 12 cytokines, eight transcription factors and 24 key molecules. This map shows how the cytokines and transcription factors considered in this study interact with 24 key molecules. The key molecules, JUN, FOS and FOSB are represented with AP-1. Another key molecule, IL2RG is represented with IL-21R.

We have measured the four centralities of all the nodes present in the network and plotted their histograms (Fig 2). With the histograms as reference, we have extracted approximately 20% of the nodes with the highest centralities in each category. The degree distributions of scale free networks, such as many real networks and human PPI, have a power-law tail. As a consequence of this, a few highly connected nodes exist in the whole network. Researchers generally refer to the 20% of nodes with the highest degree in a network as the hubs [50]. The degree and eigenvector histograms have a power-law tail (Fig 2). We extended top 20% nodes to the betweenness and closeness centrality categories in order to increase the number of central nodes. This resulted in ~30% (354) of the total nodes in CPPIN as the central nodes. Then we proceeded to determine how many of these nodes are differentially expressed in the RA synovium. The nodes that are selected in (a) at least three of the four centrality measures and differentially expressed in three of the five microarray datasets or (b) that are selected in at least two centrality measures and differentially expressed in at least four microarray datasets were considered the key molecules. This resulted in a total of 24 molecules (Table 3). A concise interaction map that shows how these molecules are related to 12 cytokines and eight transcription factors, and among them, is shown in Fig 3. This map can also be visualized in cytoscape using the S6 File.

**Table 3:** Centrality and differential expression of the CPPIN network proteins. The proteins that are selected in at least (a) three centrality measures and three synovial microarray datasets or (b) two centrality measures and four synovial microarray datasets are considered as the key molecules. This resulted in 24 key molecules.

S.No.	Key molecule	Degree	Betweenness	Closeness	Eigenvector	Number of microarray datasets in which the gene is differentially expressed				
						1	2	3	4	5
1	SYK									
2	PTPN6									
3	LCK									
4	PTPRC									
5	INPP5D									
6	PRKCB									
7	CD3E									
8	CSF2RB									
9	IL2RG									
10	EGFR									
11	JUN									
12	MYC									
13	FOS									
14	AR									
15	CCR5									
16	IFNAR2									
17	LRP1									
18	MX1									
19	LAP3									
20	CXCR4									
21	EGR1									
22	FOSB									
23	TNFRSF14									
24	CD48									

Blue color indicates the presence of the protein in the top 20% centrality list. Green color indicates the up-regulation and red color indicates the down-regulation.

### Directionality of differential expression for cytokine-transcription factor shortest path molecules

**Fig 4:**
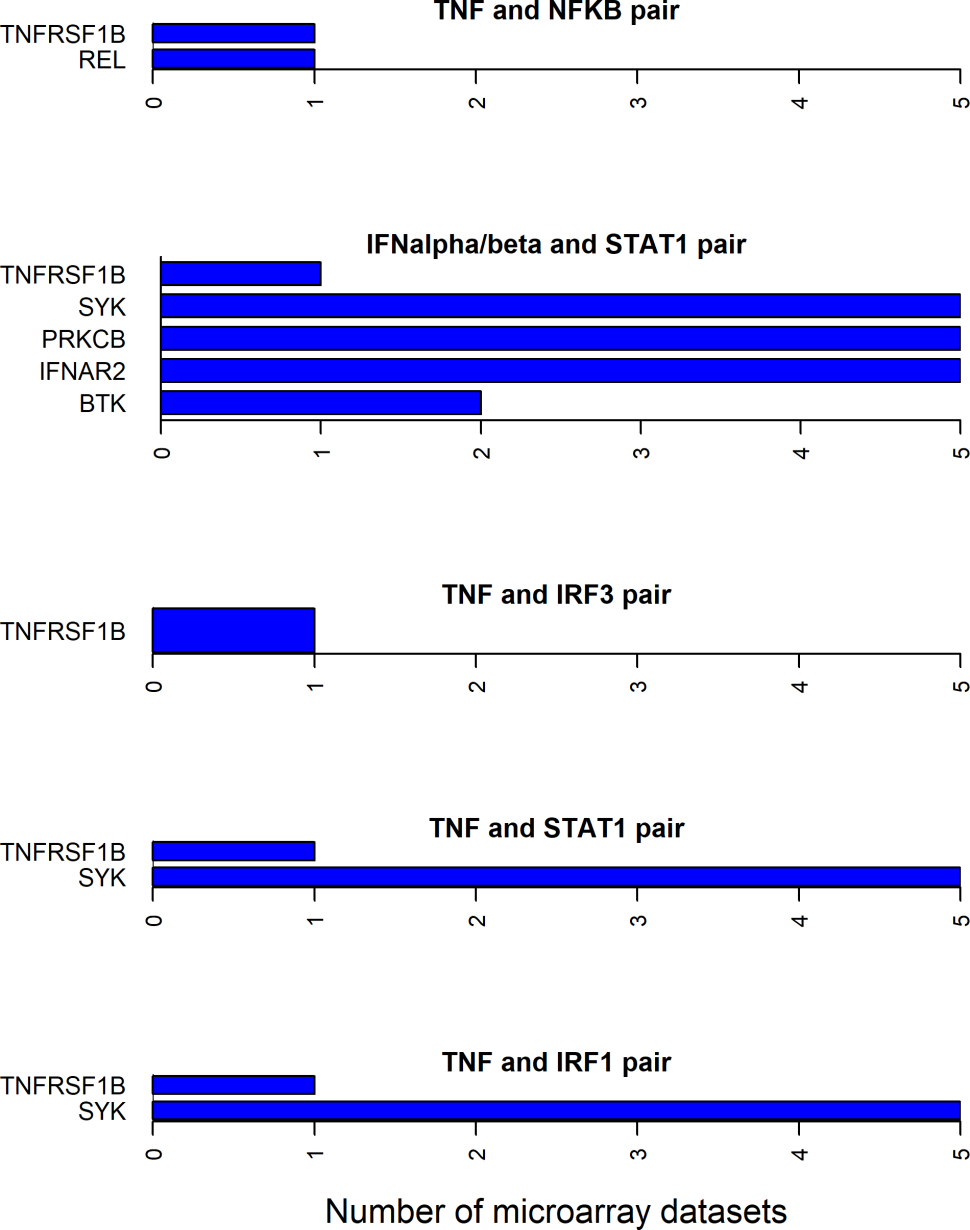
Exclusively up-regulated shortest path molecules for TNF and IFNα/β related pathways. The figure shows the completely up-regulated shortest path molecules in TNF and NF-κB, IRF3, IRF1 and STAT1 pairs, and IFNα/β and STAT1 pair.

**Fig 5:**
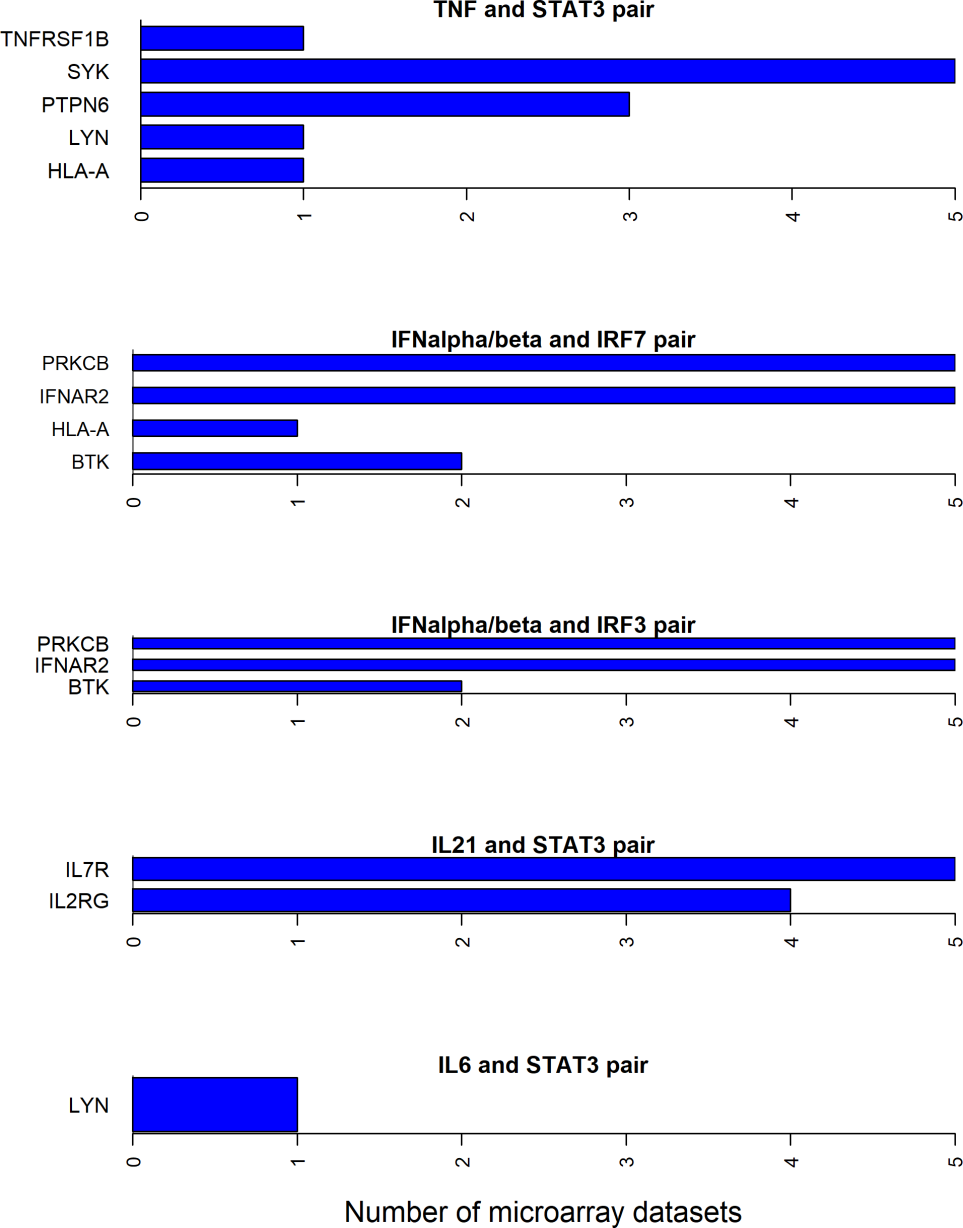
Exclusively up-regulated shortest path molecules for TNF and IFNα/β, IL-21 and IL6 related pathways. The figure shows the completely up-regulated shortest path molecules in TNF, IL-21 and IL6 cytokines and STAT3 transcription factor pairs, and IFNα/β cytokine and IRF3 and IRF7 pairs.

**Fig 6:**
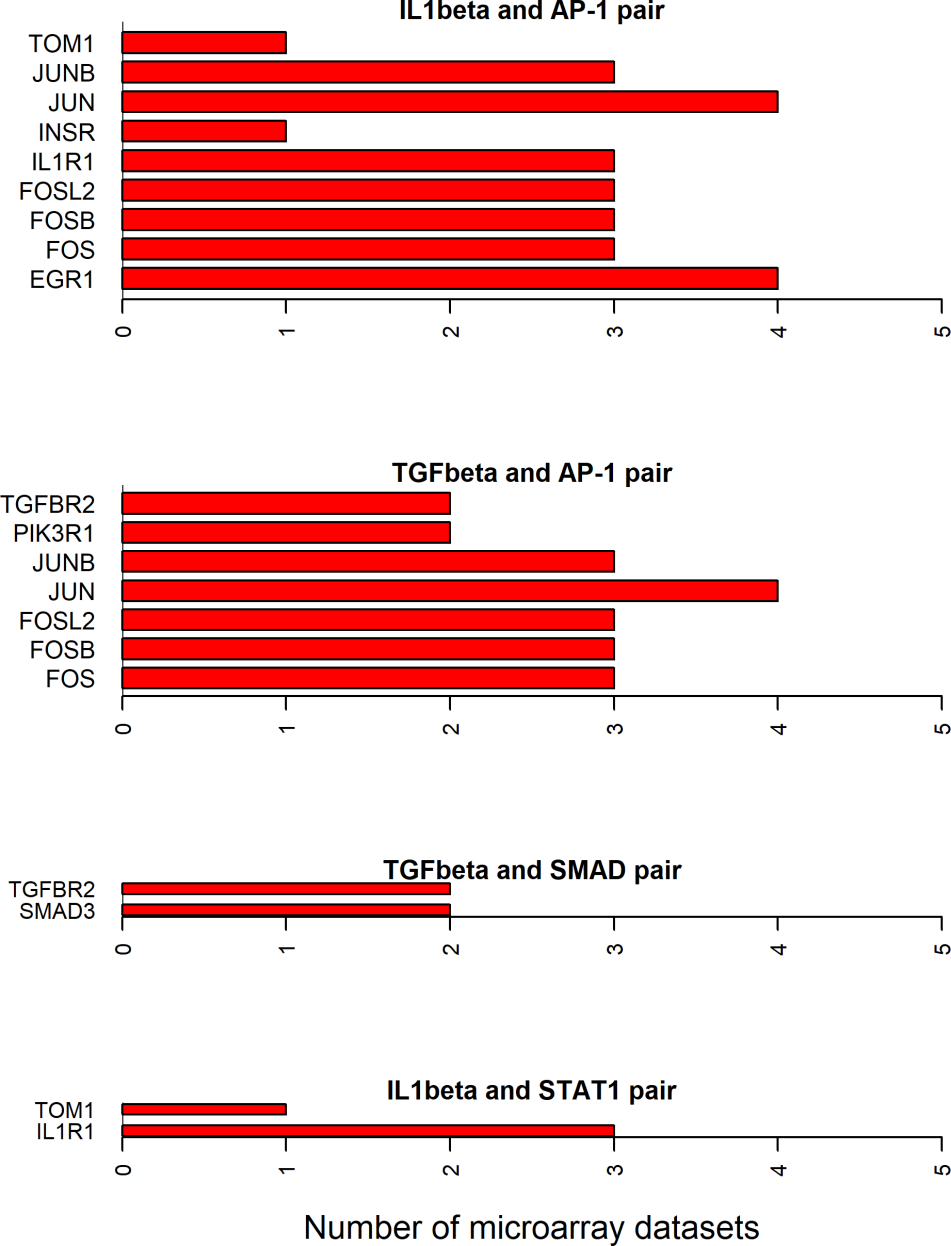
Exclusively down-regulated shortest path molecules for IL-1β and TGF-β related pathways. The figure shows the completely down-regulated shortest path molecules in IL-1β and TGF-β cytokines and AP-1, SMAD and STAT1 transcription factor pairs.

**Fig 7:**
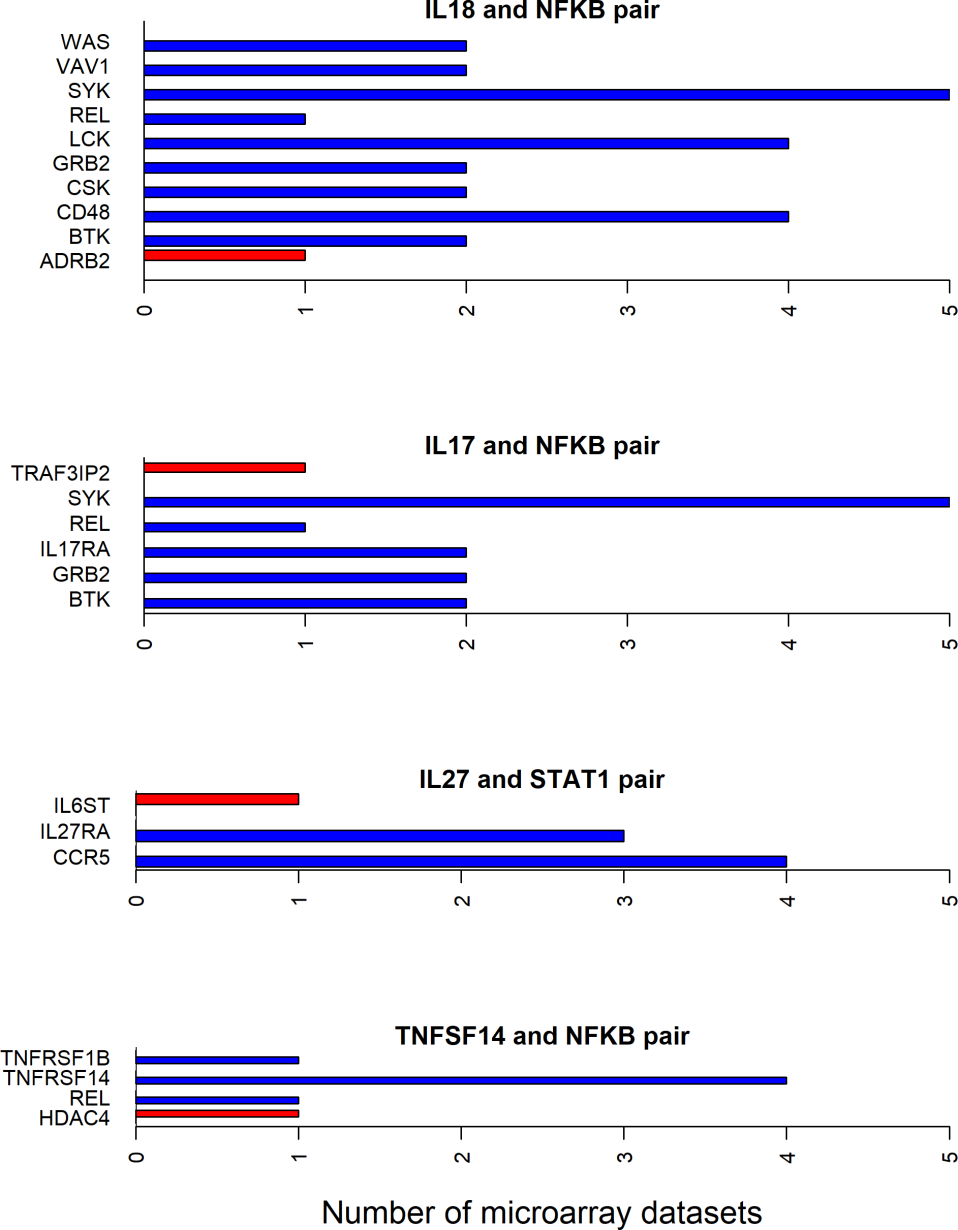
Mostly up-regulated shortest path molecules in IL-18, IL-17, TNFSF14 and IL-27 related pathways. The figure shows the mostly up-regulated shortest path molecules in IL-18, IL-17 and TNFSF14 cytokines and NF-κB transcription factor pairs, and IL-27 and STAT1 pair. The blue and red bars represent the up- and down-regulated genes respectively.

**Fig 8:**
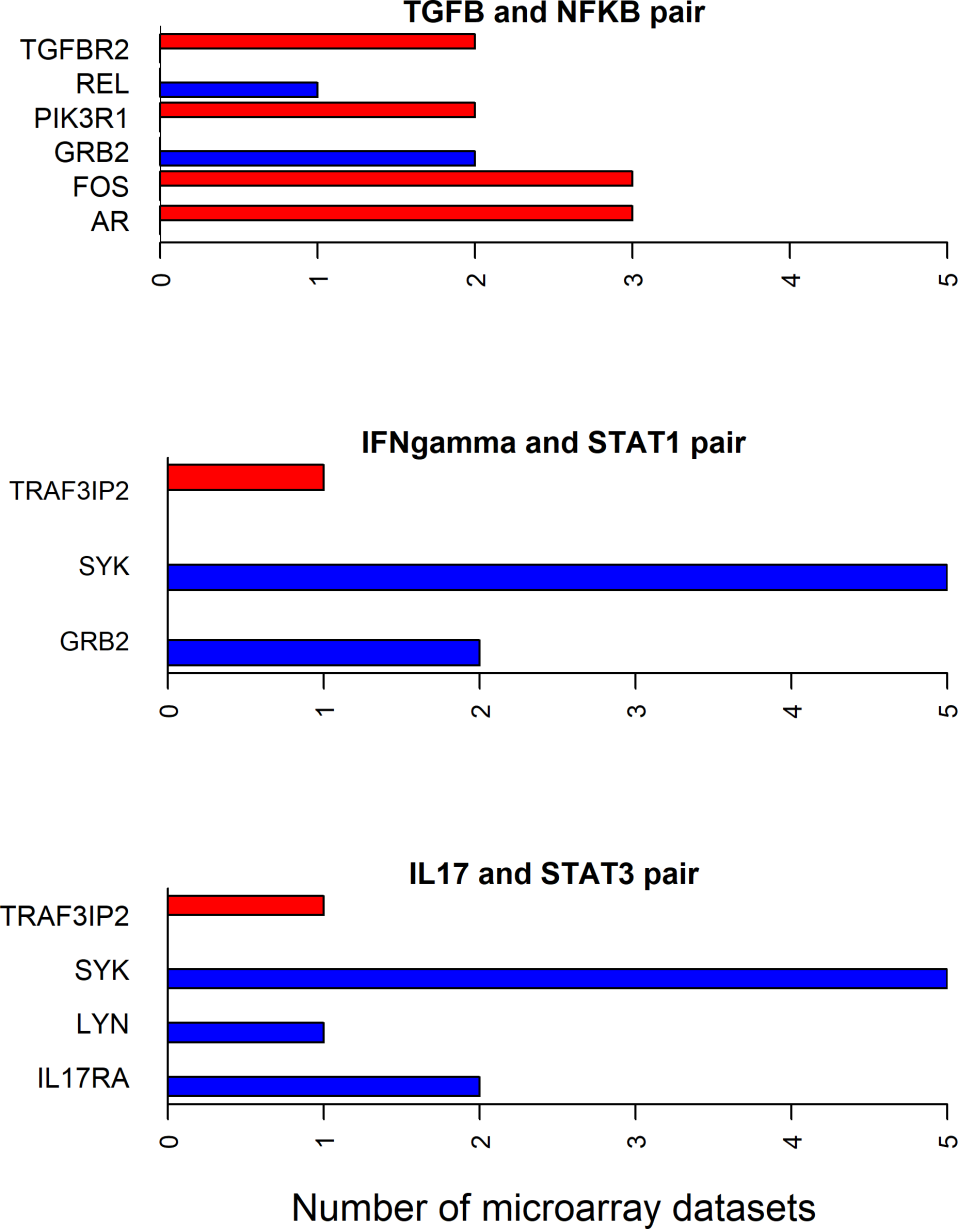
Up- and down-regulated shortest path molecules in IFNγ, IL-17 and TGF-β related pathways. The figure shows the mostly up-regulated shortest path molecules in IFNγ and STAT1, IL-17 and STAT3 pairs. It also shows the mostly down-regulated molecules in the shortest path between TGF-β and NF-κB pair. The blue and red bars represent the up- and down-regulated genes respectively.

**Fig 9:**
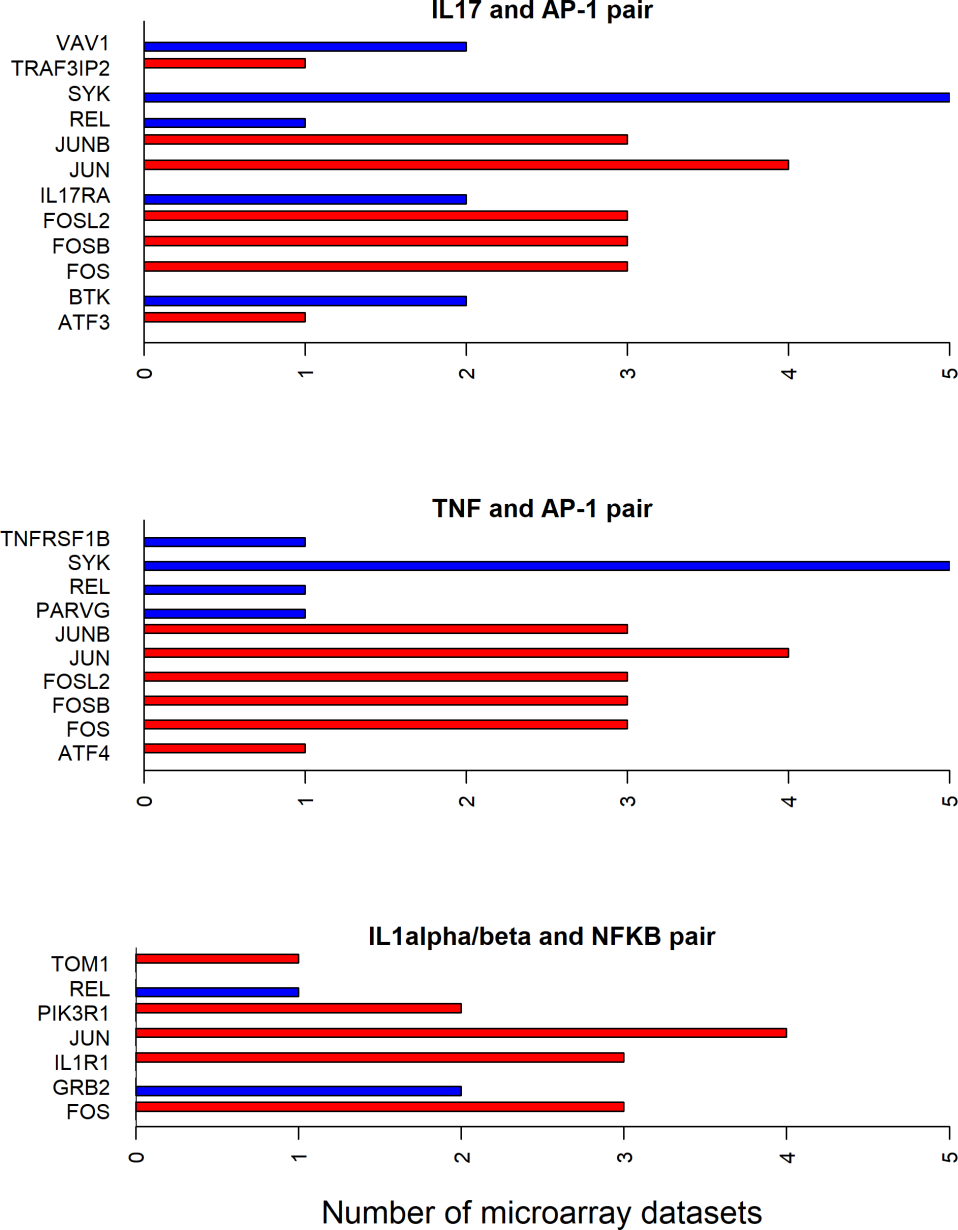
Mostly down-regulated shortest path molecules in IL-17, TNF and IL-1α/β related pathways. The figure shows the mostly down-regulated shortest path molecules in IL-17 and TNF cytokines and AP-1 transcription factor pairs, and IL-1α/β and NF-κB pair. The blue and red bars represent the up- and down-regulated genes respectively.

**Fig 10:**
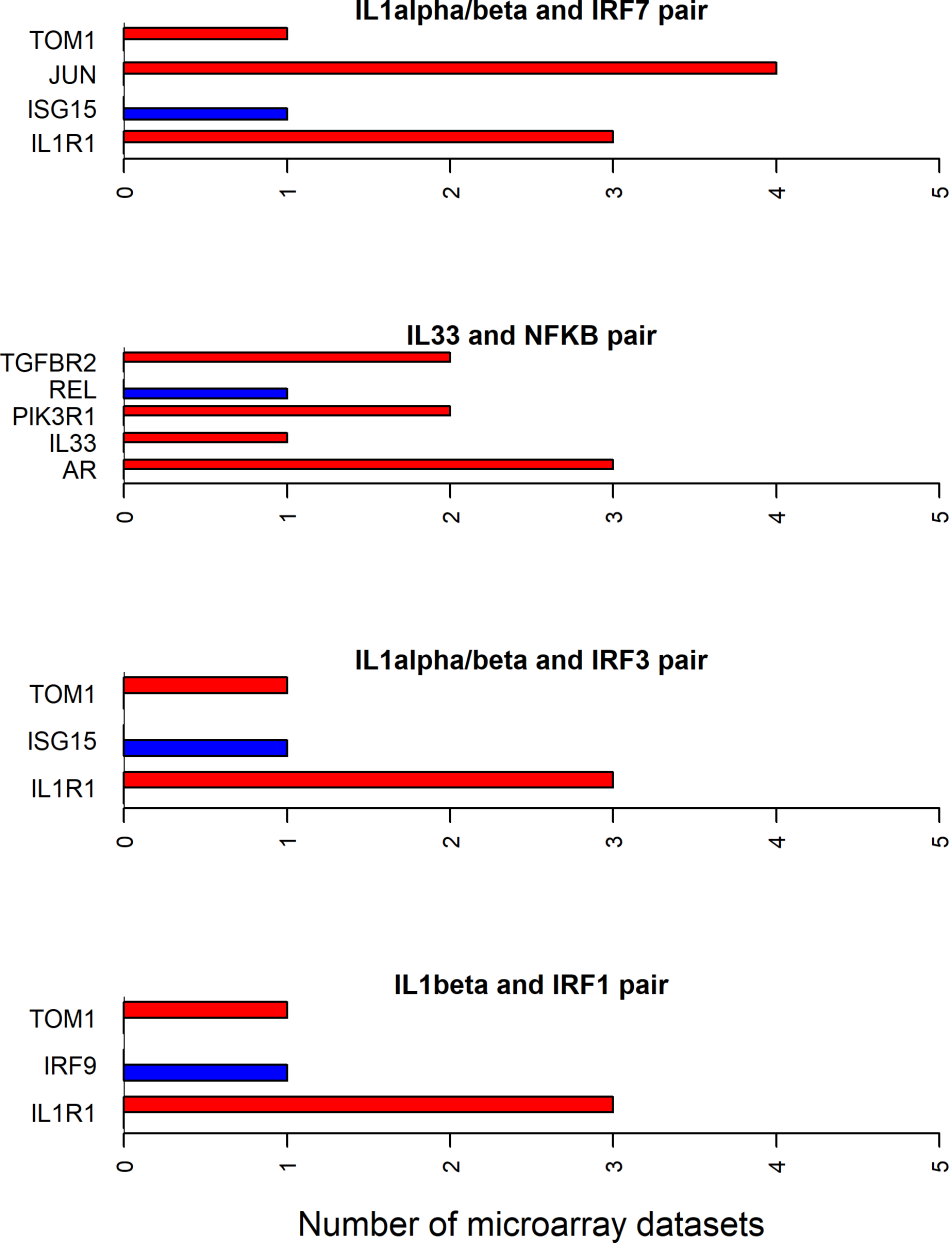
Mostly down-regulated shortest path molecules in IL-1α/β and IL-33 related pathways. The figure shows mostly down-regulated shortest path molecules in IL-1α/β cytokine and IRF7, IRF3 and IRF1 transcription factor pairs, and IL-33 and NF-κB pair. The blue and red bars represent the up- and down-regulated genes respectively.

Since all the 12 cytokines are known to induce signal transduction pathways in RASF, all the shortest paths—between a given cytokine and transcription factor pair—seem to be important. However, some of these paths have the molecules with the same directionality (up- or down-regulated) of differential expression between RA and normal samples in synovial microarray datasets (Figs 4–6). Some cytokine and transcription factor pair shortest paths have higher number of up-regulated molecules over down-regulated molecules (Figs 7 and 8). Some other paths have higher number of down-regulated molecules over up-regulated molecules (Figs 8–10). From this we can conclude that all these pathways are getting affected in RA. Some are active because they have a high proportion of up-regulated molecules; some others are inactive because they have a high proportion of down-regulated molecules; few of them are dysregulated because they have both up- and down-regulated molecules.

### Effects of medical therapy initiation on gene expression

**Fig 11:**
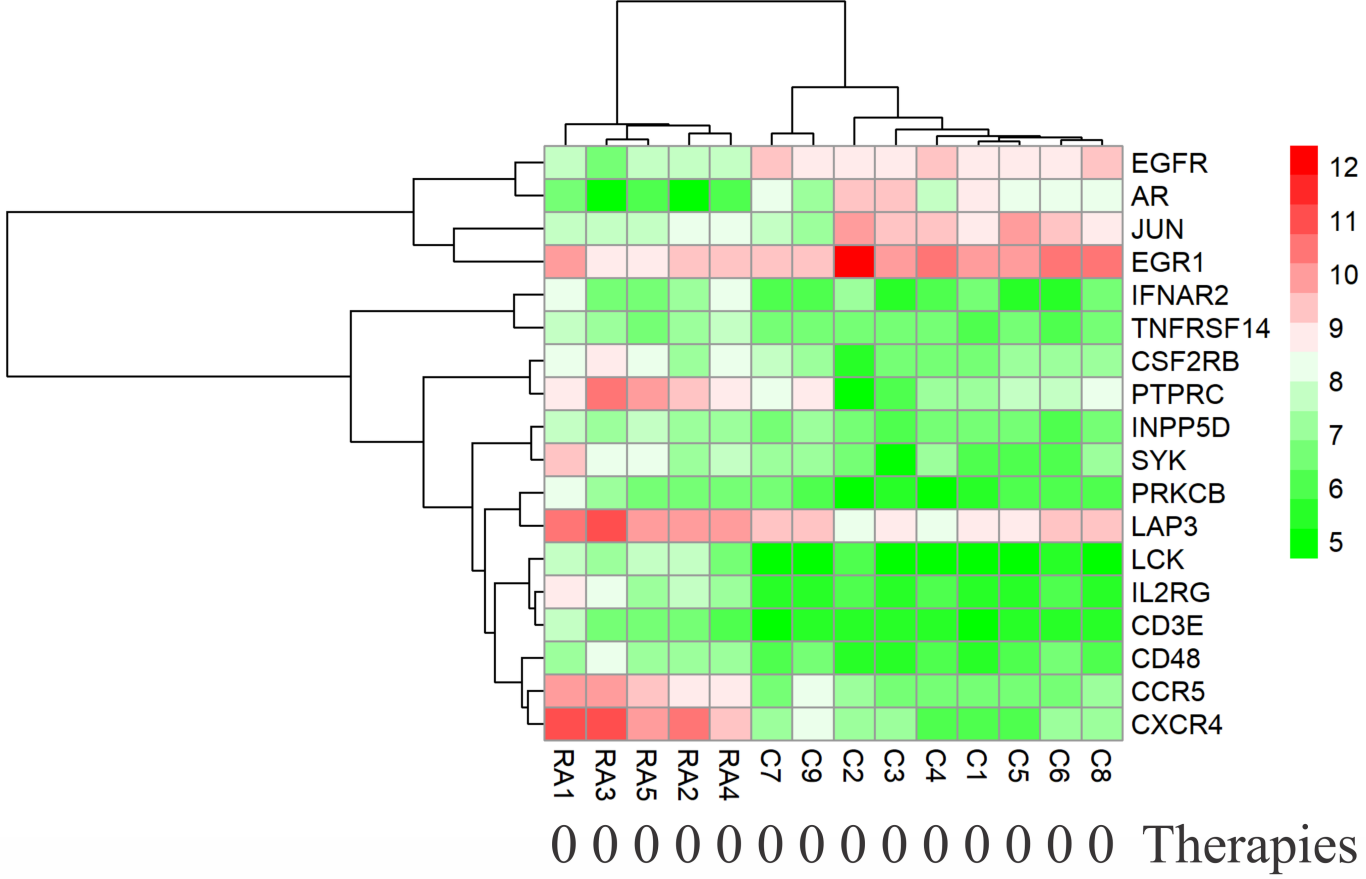
Hierarchical clustering of RA and control samples based on the gene expression of selected key molecules in GSE7307. The RA patients belonging to this dataset were not treated with drugs. The RA and control samples were clustered into separate groups.

**Fig 12:**
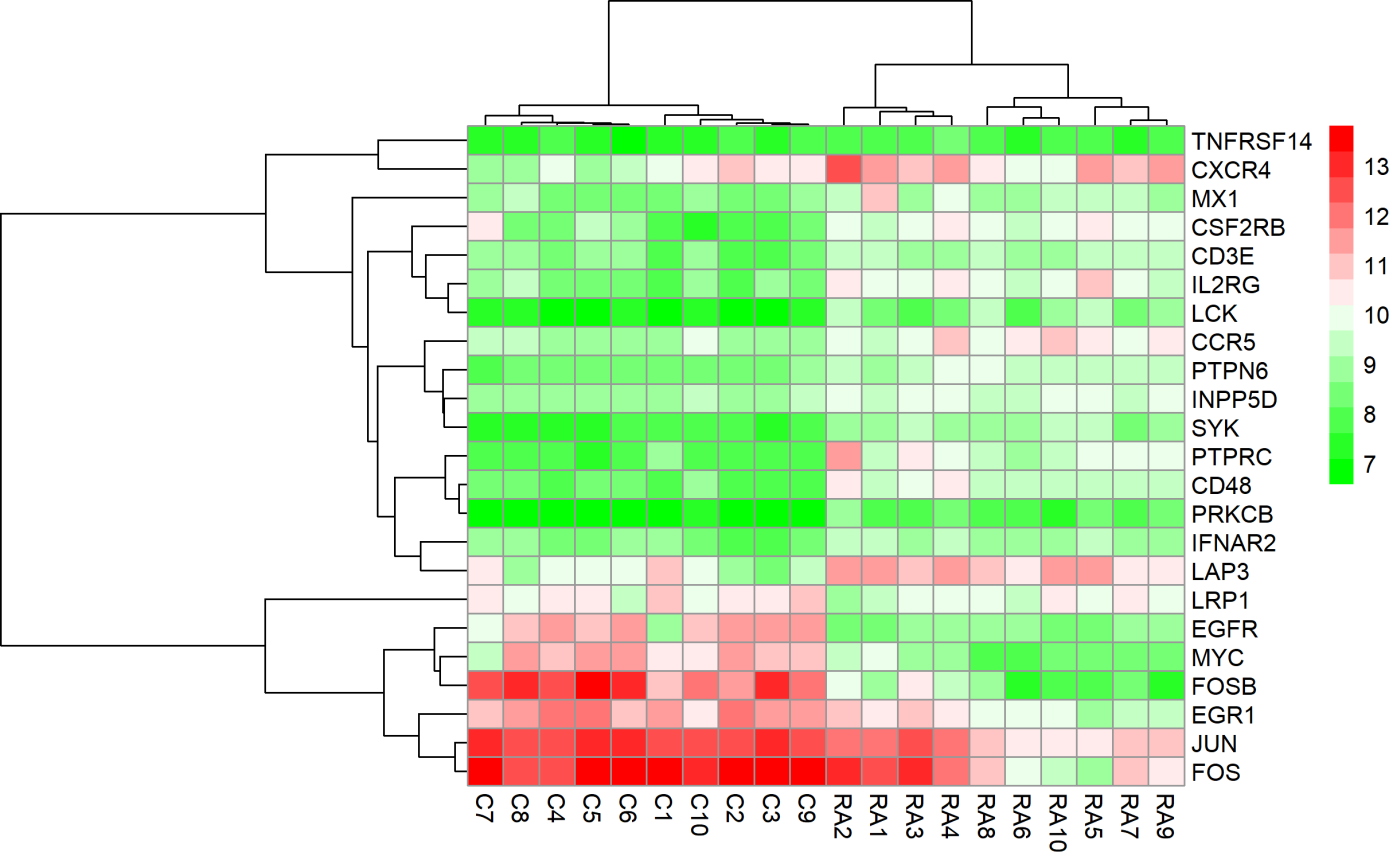
Hierarchical clustering of RA and control samples based on the gene expression of selected key molecules in GSE55235. The RA and control samples were clustered into separate groups.

**Fig 13:**
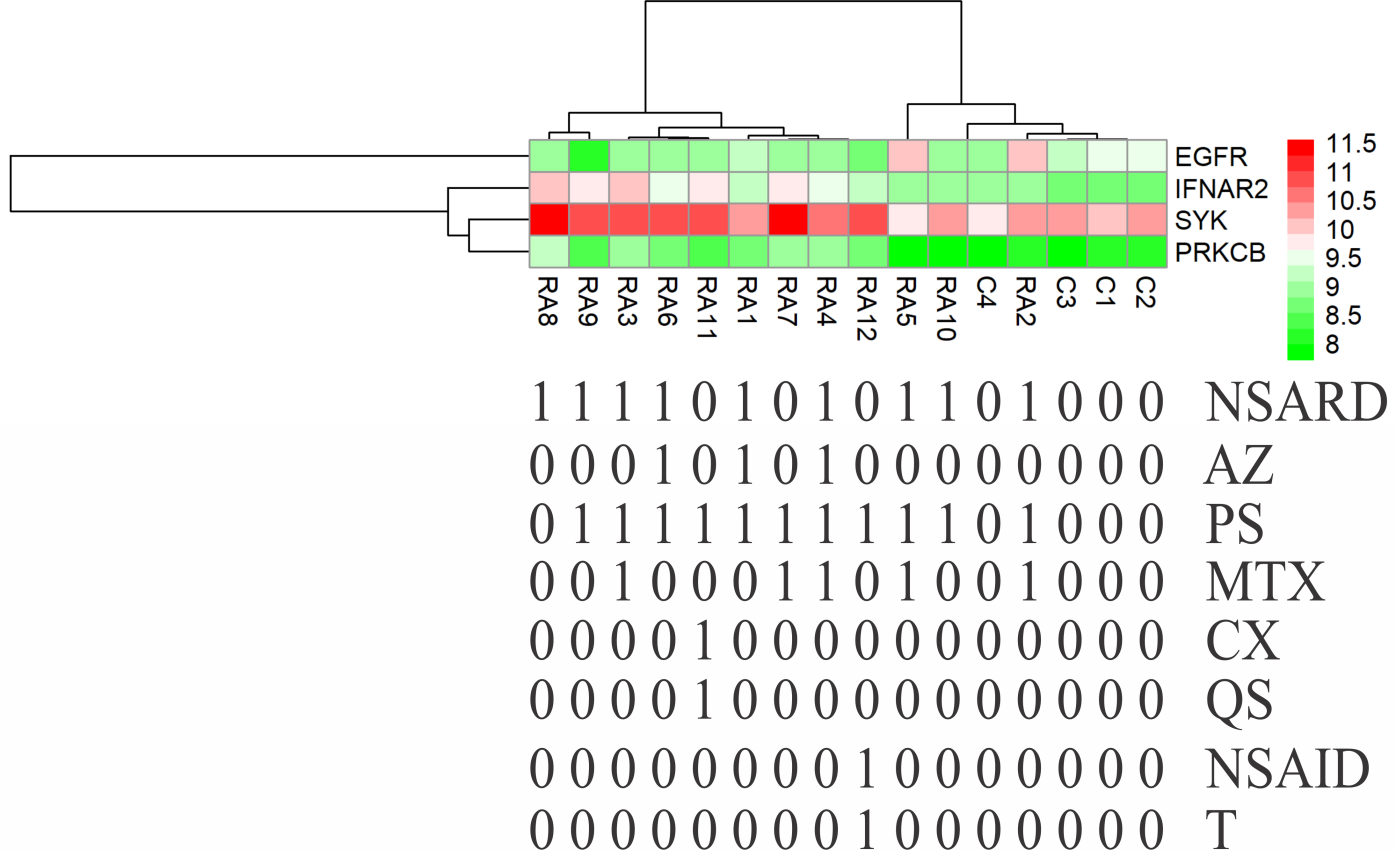
Hierarchical clustering of RA and control samples based on the gene expression of selected key molecules in GSE12021 (HGU133B). The RA (except one) and control samples were clustered into separate groups. The figure also shows the combinations of drugs used for treating RA patients.

**Fig 14:**
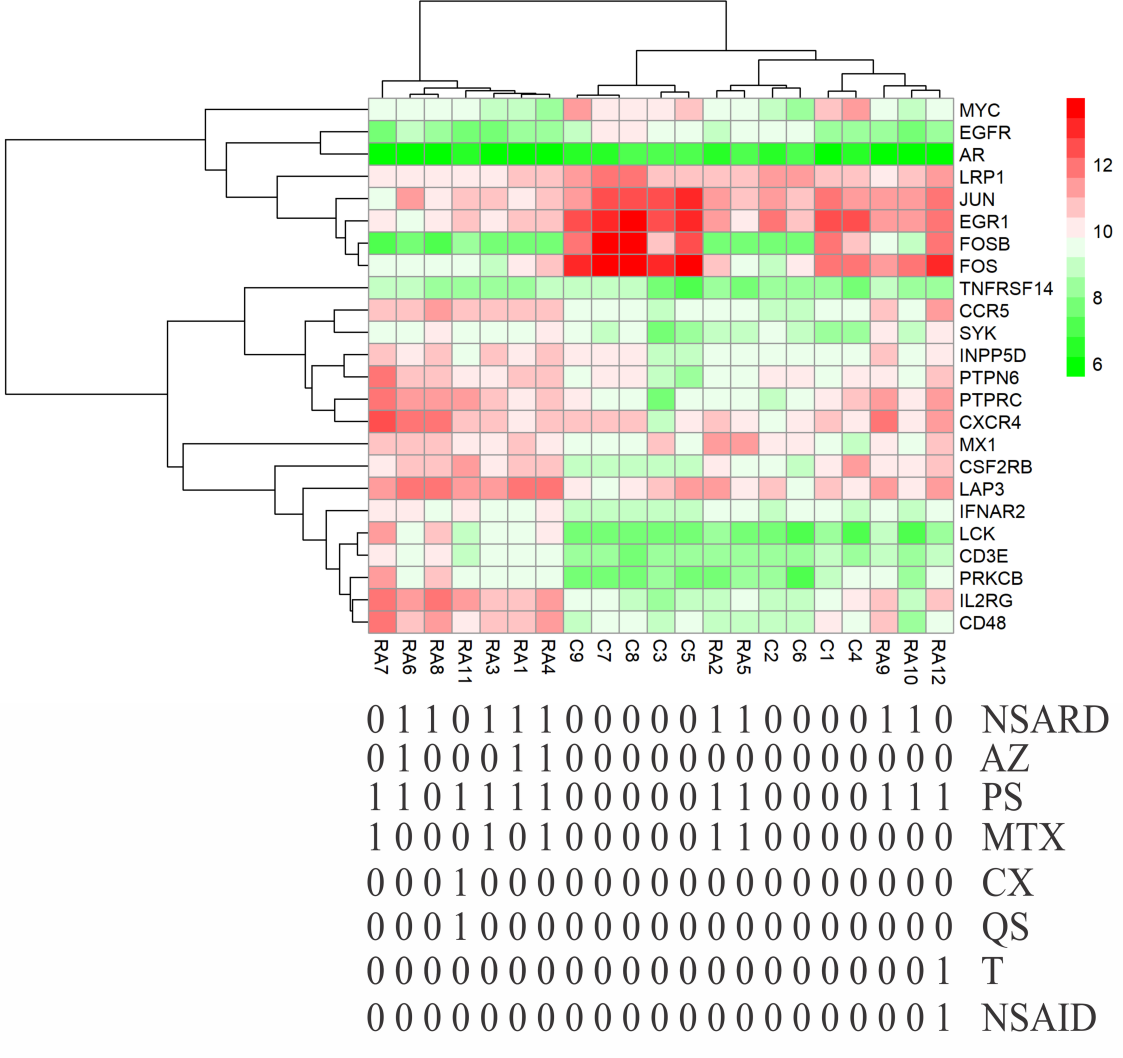
Hierarchical clustering of RA and control samples based on the gene expression of selected key molecules in GSE12021 (HGU133A). Some RA samples were clustered into a separate group while others were clustered with control samples. The figure also shows the combinations of drugs used for treating RA patients.

**Fig 15:**
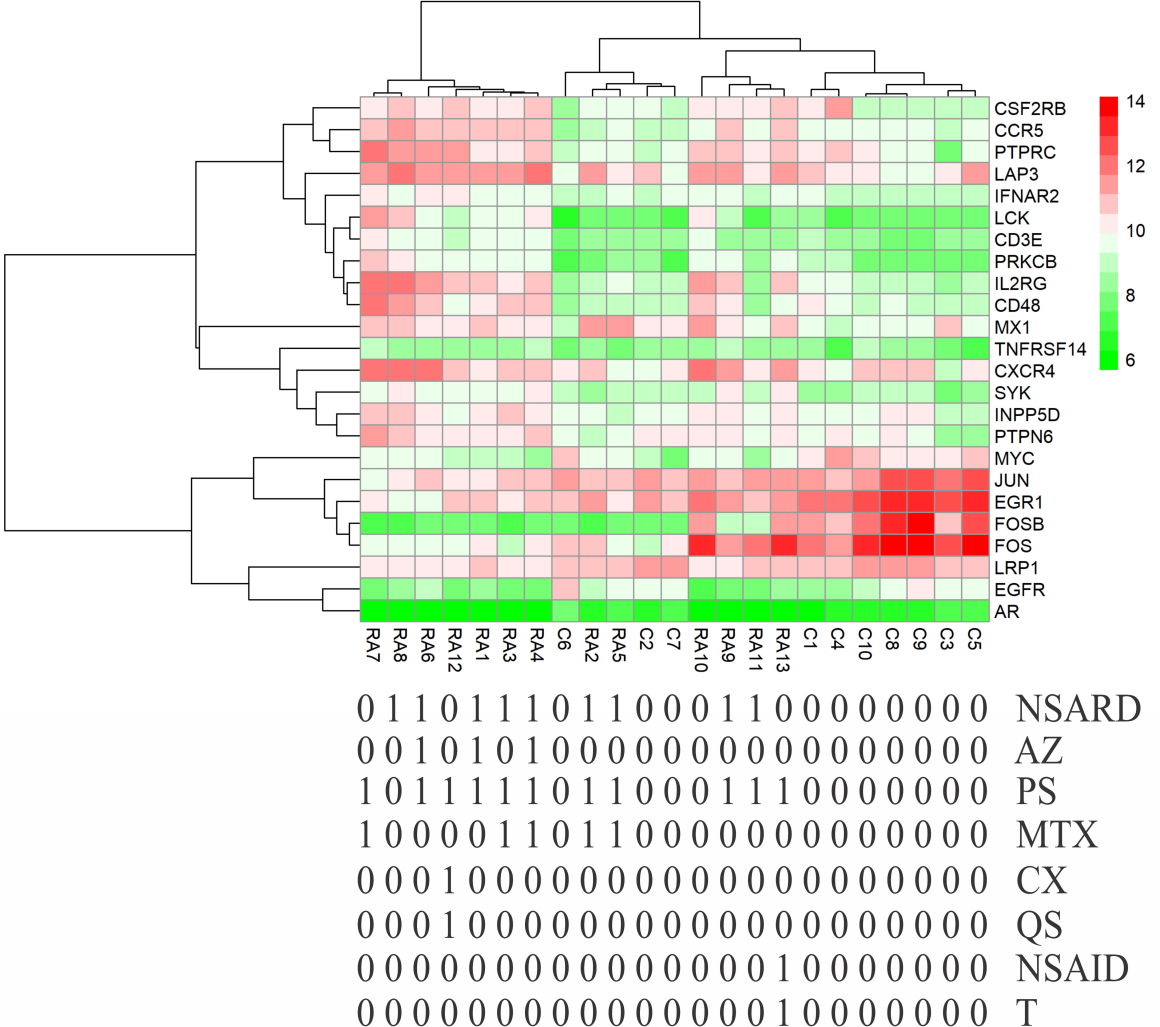
Hierarchical clustering of RA and control samples based on the gene expression of selected key molecules in GSE55457. Some RA samples were clustered into a separate group while others were clustered with control samples. The figure also shows the combinations of drugs used for treating RA patients.

Except for GSE7307 and GSE55235, each RA patient belonging to other microarray studies considered in this study has undergone different combinations of medical therapies. The details of therapies are in Table 4. The therapies initiated on the patients are non-steroidal anti-rheumatic drug (NSARD), Azulfidine (AZ), Prednisolone (PS), Methotrexate (MTX), Cox-2 inhibitor (CX), Quensyl (QS), non-steroidal anti-inflammatory drug (NSAID) and Tilidin (T). Some patients within a dataset were treated with the same combination of therapies while others were treated with different combinations. To find out how these therapies could have affected the gene expression of 24 key molecules, the samples in each dataset were hierarchically clustered based on the expression values. In GSE7307, GSE55235 and except for one RA sample in GSE12021 (HGU133B), all RA and control samples were clustered into separate groups (Figs 11–13). In GSE12021 (HGU133A) and GSE55457, some RA samples were clustered into a separate group while others were clustered with controls (Figs 14 and 15), showing that there is a drug effect.

**Table 4:** Medical therapies initiated on RA patients.

Dataset	Patients	Medical Therapies
GSE7307	RA1	Not treated
	RA2	Not treated
	RA3	Not treated
	RA4	Not treated
	RA5	Not treated
GSE12021 (HGU133A)	RA1	NSARD + Azulfidine + Prednisolone
	RA2	NSARD + MTX + Prednisolone
	RA3	NSARD + MTX+ Prednisolone
	RA4	NSARD + Azulfidine + Prednisolone + MTX
	RA5	NSARD + MTX + Prednisolone
	RA6	NSARD + Azulfidine + Prednisolone
	RA7	MTX + Prednisolone
	RA8	NSARD
	RA9	NSARD + Prednisolone
	RA10	NSARD + Prednisolone
	RA11	COX-2 inhibitor + Prednisolone + Quensyl
	RA12	NSAID + Tilidin + Prednisolone
GSE12021 (HGU133B)	RA1	NSARD + Azulfidine + Prednisolone
	RA2	NSARD + MTX + Prednisolone
	RA3	NSARD + MTX+ Prednisolone
	RA4	NSARD + Azulfidine + Prednisolone + MTX
	RA5	NSARD + MTX + Prednisolone
	RA6	NSARD + Azulfidine + Prednisolone
	RA7	MTX + Prednisolone
	RA8	NSARD
	RA9	NSARD + Prednisolone
	RA10	NSARD + Prednisolone
	RA11	COX-2 inhibitor + Prednisolone + Quensyl
	RA12	NSAID + Tilidin + Prednisolone
GSE55457	RA1	NSARD + Azulfidine + Prednisolone
	RA2	NSARD + MTX + Prednisolone
	RA3	NSARD + MTX+ Prednisolone
	RA4	NSARD + Azulfidine + Prednisolone + MTX
	RA5	NSARD + MTX + Prednisolone
	RA6	NSARD + Azulfidine + Prednisolone
	RA7	MTX + Prednisolone
	RA8	NSARD
	RA9	NSARD + Prednisolone
	RA10	no therapy used
	RA11	NSARD + Prednisolone
	RA12	COX-2 inhibitor + Prednisolone + Quensyl
	RA13	NSAID + Tilidin + Prednisolone
GSE55235	RA1	Therapies not mentioned
	RA2	Therapies not mentioned
	RA3	Therapies not mentioned
	RA4	Therapies not mentioned
	RA5	Therapies not mentioned
	RA6	Therapies not mentioned
	RA7	Therapies not mentioned
	RA8	Therapies not mentioned
	RA9	Therapies not mentioned
	RA10	Therapies not mentioned

In order to find the effect of medical therapies on the differential expression of genes, we removed the RA samples that were clustered with healthy controls from GSE12021 (HGU133A), GSE12021 (HGU133B) and GSE55457 datasets and repeated the differential expression analysis for the 354 genes which encode central proteins of CPPIN. With the same selection criteria of differential expression and centrality measures, all the 24 genes of key molecules were retained, and in addition, 10 other genes that encode central proteins were also selected (S1 Table). S1–S3 Figs show the heat maps of the expression levels of these 24 genes in these three data sets after eliminating the RA samples that clustered with healthy controls. We notice the complete separation of controls from RA samples in the clusters.

We conclude that the 24 genes that were differentially expressed in both the cases were not affected by the therapy initiation while the 10 other genes that were selected in the second case might have been affected.

### Direct PPI links of the CPPIN key molecules with current RA drug targets

**Fig 16:**
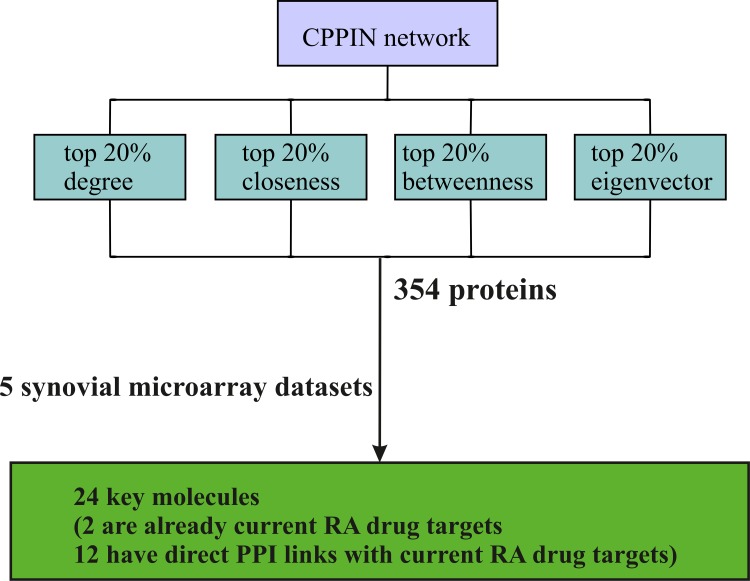
The overall strategy used for identifying the key molecules. From the CPPIN network, approximately 20% of the proteins with high scores in each centrality category were extracted. This resulted in 354 proteins. The differential expression of the genes which encode these proteins was computed in five synovial microarray datasets. Finally, the genes which are selected in at least three centrality measures and three microarray datasets or two centrality measures and four microarray datasets were considered the key molecules. This resulted in 24 key molecules. Two of these, SYK and JUN, are already current RA drug targets. Among the remaining, 12 have direct PPI links to some of the current RA drug targets.

In order to determine if there are any recognized RA drug targets in the CPPIN key molecules, we have downloaded a dataset from the Therapeutic Target Database (TTD) which contains information on drug target genes that are at various stages of the drug discovery process [80]. The dataset consists of successful drug targets as well as the ones that have been studied in research projects and clinical trials for several diseases. We extracted all the drug targets of RA from this dataset and combined them with the successful RA drug targets listed in Okada et al. [81]. The resulting ensemble of 48 RA drug targets is listed in Table 5. Two of the key CPPIN molecules, spleen tyrosine kinase (SYK) and c-Jun (JUN) are already established drug targets for RA. Among the remaining key CPPIN molecules, 12 have direct PPI links to some of the current RA drug targets (Table 6). So these 24 molecules can be considered potential drug targets for RA. The overall strategy that was used to come up with the key molecules is explained in Fig 16.

**Table 5:** An ensemble of current RA drug targets. These are the current RA drug targets that are obtained from two sources namely TTD and a scientific article, Okada et al. [81].

Drug target	Status of the target	Source
IKBKB	Clinical trial	TTD, Okada et al.
CHUK	Clinical trial	TTD
CFLAR	Research project	TTD
JUN	Clinical trial	TTD
ITGB1	Clinical trial	TTD
IL6R	Clinical trial	TTD, Okada et al.
FGF2	Clinical trial	TTD
CCL2	Clinical trial	TTD
ITGA4	successful	TTD
OSM	Research project	TTD
MIF	Clinical trial	TTD
IL1R1	successful	TTD, Okada et al.
LIF	Research project	TTD
MMP8	successful	TTD
IL13	Clinical trial	TTD
PTGS2	successful	TTD, Okada et al.
IL6ST	Clinical trial	TTD
IL15	Clinical trial	TTD
CTSK	successful	TTD
SYK	Clinical trial	TTD
JAK3	successful	TTD, Okada et al.
MAPK12	Clinical trial	TTD
F2RL1	Research project	TTD
IL2	Clinical trial	TTD
DHODH	successful	TTD, Okada et al.
IKBKE	Clinical trial	TTD
MYD88	Research project	TTD
TLR9	Clinical trial	TTD, Okada et al.
TNF	successful	Okada et al.
CD80	successful	Okada et al.
CD86	successful	Okada et al.
MS4A1	successful	Okada et al.
PRDX5	successful	Okada et al.
HPRT1	successful	Okada et al.
CAMLG	successful	Okada et al.
PPP3R2	successful	Okada et al.
ELANE	successful	Okada et al.
DHFR	successful	Okada et al.
ALOX5	successful	Okada et al.
PTGS1	successful	Okada et al.
PPARG	successful	Okada et al.
FKBP1A	successful	Okada et al.
MTOR	successful	Okada et al.
JAK1	successful	Okada et al.
JAK2	successful	Okada et al.
NR3C1	successful	Okada et al.
NR3C2	successful	Okada et al.
TLR7	successful	Okada et al.

TTD, therapeutic target database.

**Table 6:** Direct PPI links of the key molecules with current RA drug targets.

S.No.	Key molecule	Direct PPI interaction with the RA drug targets
1	PTPN6	JAK1, JAK2, SYK
2	LCK	MS4A1, SYK
3	PTPRC	JAK2
4	INPP5D	JAK1, SYK
5	CD3E	SYK
6	CSF2RB	JAK2
7	IL2RG	IL2
8	MYC	CHUK, JUN
9	FOS	JUN
10	CCR5	JAK1, JAK2
11	EGR1	JUN
12	FOSB	JUN

### Significance of overlap of key molecules with current RA drug targets

A random sample of 24 proteins was drawn from the CPPIN network and estimated their overlap with the current RA drug targets. After removing the overlaps from the 24, for the remaining proteins we determined the direct PPI links to the drug targets. We got the following results based on one million random samples:

1. The probability of getting 2 or more drug targets out of 24 randomly selected proteins is 0.0478, giving a statistical significance of less than 5%.
2. After removing overlaps, we counted the number of proteins among the remaining ones that are directly connected to drug targets in CPPIN. The probability of getting 12 or more proteins with direct connections to drug targets is found to be 2.6×10^−5^.

Thus, the selection of 24 proteins by our analysis with two drug targets and at least 12 direct PPI links to drug targets has statistical significance.

## Discussion

In this study, we have built a cytokine signaling network (CPPIN) in RASF using publicly available PPI data. The CPPIN network contains 12 cytokine pathways that are active in RASF. The cytokine receptors, their transcription factors, intermediate signal transducers that connect them and the direct interacting proteins of the intermediates are part of this network. The cytokines include TNF, interleukins—IL-1α/β, IL-6, IL-17, IL-18, IL-21, IL-27 and IL-33, tumor necrosis factor superfamily member 14 (TNFSF14), three interferons (IFNα/β and IFNγ) and transforming growth factor-beta1 (TGF-β1). The transcription factors include nuclear factor kappa-light-chain-enhancer of activated B cells (NF-κB), signal transducer and activator of transcription 1 (STAT1), STAT3, activator protein 1 (AP-1), interferon regulatory factor 1 (IRF1), IRF3, IRF7 and SMAD. Even though the number of cytokines considered in this study is low, they are reliable in the sense that they stimulate RASF and induce signal transduction pathways leading to the activation of their respective transcription factors.

For building this comprehensive network, we have considered the PPI interactions only if the interacting participants of each interaction are co-expressed in the synovial tissues. To find the central proteins of the CPPIN network, four centrality measures, degree, closeness, betweenness and eigenvector have been measured for all the proteins of the CPPIN network. In each centrality category, approximately 20% of the proteins with high centrality scores were pulled out and the lists were merged for further analysis.

To identify the differential expression of the genes that encode the proteins with high centrality values we analyzed five microarray datasets related to RASF in the GEO database. We have used the following methodology to come up with the key molecules. A protein is considered a key molecule only if it is selected in at least three centrality measures and three microarray datasets or two centrality measures and four microarray datasets. This gave 24 key molecules. Two of these 24, namely JUN and SYK, are already drug targets for RA. Among the remaining, 12 have direct PPI links to current RA drug targets.

One of the key molecules in the list is epidermal growth factor receptor (EGFR). EGFR is down-regulated in at least five RA microarray datasets. It is also highly connected in the CPPIN network as it has high degree, betweenness and closeness centrality measures. Swanson et al. observed that inhibition of EGFR by erlotinib reduced pannus formation, synovitis, vascularisation, and cartilage and bone erosion in type-II collagen-induced arthritis (CIA) mouse models [82–83]. Further, an earlier topological analysis of a PPI network in RA has reported that EGFR is highly relevant to RA (Tieri et al.) [84]. Another key molecule, tyrosine-protein phosphatase non-receptor type 6 (PTPN6) is up-regulated in three microarray datasets and is selected in all the four centrality measures. It also interacts with some of the current RA drug targets, janus kinase 1 and 2 (JAK1 and JAK2) and SYK. Additionally, it is found to be enriched in the synovial fluid of RA patients [85].

Lymphocyte-specific protein tyrosine kinase (LCK) is up-regulated in four microarray datasets. It is also selected in all the four centrality measures. Further, Swanson et al. have affirmed that tyrosine kinases such as LCK are the predominant players in the cell signaling pathways that enhance inflammation and the formation of pannus in RA. They emphasized that LCK can be considered a drug target for RA [86]. Another key molecule known as colony stimulating factor 2 receptor beta (CSF2RB) is up-regulated in four microarray datasets and is selected in three centrality measures. It interacts with an RA drug target JAK2. Additionally, Fujikado et al. have observed the up-regulation of the CSF2RB gene in their mouse RA models [87]. Interleukin receptor subunit gamma (IL2RG), which interacts with at least six cytokine receptors including IL2RA, IL4RA, IL7RA, IL9RA, and IL15RA, is up-regulated in four microarray datasets and is selected in all the four centralities. In addition, Chang et al. have observed the up-regulation of IL2RG in their microarray studies on RA synovial tissues [88]. c-myc (MYC) is down-regulated in three microarray datasets and is selected in all the four centralities. It also interacts with two current RA drug targets, the conserved helix-loop-helix ubiquitous kinase (CHUK) and JUN. Hashiramoto et al. have found the involvement of c-myc in RA pathogenesis [89]. They have observed that c-myc antisense oligodeoxynucleotides (AS ODN) arrested cell proliferation and induced apoptosis in rheumatoid synoviocytes. Further, Pap et al. have found c-myc, in co-operation with c-Raf-1, controls the growth and invasiveness of RASFs in the SCID mouse model of RA [90]. A subunit of the transcription factor AP-1, c-Fos (FOS), is down-regulated in three microarray datasets and is selected in all the four centrality measures. Further, Aikawa et al. have reported the suppression of RA by a small molecule inhibitor of c-Fos in CIA mice models [91]. CCR5 is up-regulated in four microarray datasets and is also selected in three centrality measures. Further, it interacts with the RA drug targets, JAK1 and JAK2. However, in a preclinical study blocking CCR5 with its antagonists and subsequently testing the effects of CCR5 blocking in a clinical trial with RA patients has not reported any clinical benefit [92–93]. The C-X-C chemokine receptor type 4 (CXCR4) is up-regulated in four microarray datasets and is selected in two centrality measures. Schmutz et al. have also observed the up-regulation of CXCR4 in synovial tissues [94]. Further, the antagonists of CXCR4 have reduced angiogenesis in the CIA murine models of RA [95]. Some antagonists of CXCR4 such as AMD3100 and a T140 analog have also reduced joint inflammation and the severity of RA [96–97]. Furthermore, low-level laser irradiation has reduced the expression of CXCR4 in CIA rat models of RA [98]. Even the single nucleotide polymorphisms (SNPs) of some genes such as PTPRC and TNFRSF14 are associated with RA [99–100].

In the current study, microarray and human PPI data were combined to generate a cytokine signaling network. We identified key molecules, which are the central proteins of this network with differential expression in RA. We also identified how these key molecules are connected to some of the current RA drug targets. Our strategy is based on a two dimensional information involving PPI and gene expression data. This network-based strategy, which led to the identification of key molecules of the cytokine signaling network, may be used for identifying multiple biomarkers, which may have potential for monitoring therapy responses.

Even though eight of the key molecules were down-regulated, their knowledge can be used for making strategies for drug discovery. For instance, designing drugs in such a way that they (i) enhance the expression of the down-regulated genes or (ii) inhibit the action or expression of a particular molecule which is known to cause the down-regulation of the key molecule (for instance, inhibition of a transcriptional repressor) can be a useful strategy in dealing with the down-regulated genes. In addition, the gene expression signatures which include both the up- and down-regulated genes can be used for screening a library of bioactive small molecules using the connectivity map (CMAP) database [101]. The molecules can further be explored for their exact targets and mechanism. This way, the gene expression signatures can essentially be used in discovering new knowledge from existing knowledge. Moreover, biological systems are robust because the perturbations caused by drug treatments can be restored. Overcoming robustness is likely to be the key factor for finding better drug targets. In this respect, both the up- and down-regulated genes may be leveraged for a multi-targeted approach.

In building the CPPIN network, we did not look for the differential expression of the cytokines in the microarray data. Cytokines are autocrine, paracrine and endocrine signaling molecules and they might be secreted by a bunch of different populated cell types in the synovium and synovial fluid of the patients with RA. Since they come from a variety of sources, they may not be differentially expressed in the microarray data. If they are secreted by other cell types and are present in the microenvironment of RASF, they can induce signaling pathways. All of the reported 12 cytokines in this study are known to be elevated in RA and they are known to activate their corresponding eight transcription factors. Elevated levels of the transcription factors may contribute to the enhanced expression of their target genes. However, an activated transcription factor may induce the expression of its target genes even though it is not differentially expressed but present in enough concentration. Therefore, we did not look for the differentially expressed cytokine and transcription factor pairs for building CPPIN.

In summary, we have built a cytokine signaling network in RA. A combination of network centrality measures and gene expression profiling data has identified 24 key molecules of this network. Two of these are already drug targets for RA while 12 others physically interact with some of the recognized drug targets. Some of these molecules such as EGFR, PTPN6, LCK, CSF2RB, IL2RG, MYC, FOS, CXCR4, PTPRC and TNFRSF14 are well studied in RA by other workers and are reported to play a role in the pathogenesis of RA. However, our strategy herein was to develop a proof-of-principle method for identifying key molecules probably involved in the pathogenesis of RA. Though some of these molecules are well studied in RA, yet their crucial involvement in the disease and their amenability for drug discovery needs to be established.

## Conclusions

The present study was focused on developing an approach that can maximize the use of the publicly available PPI and gene expression profiling data from GEO for identifying key molecules for RA. In this study, a comprehensive RA synovial-specific cytokine signaling network with 12 cytokines and 8 of their respective transcription factors has been built. Using a novel approach that combines network centrality measures and differential expression in microarray datasets, we identified 24 key molecules of this network probably involved in the pathogenesis of RA. Two of these molecules, JUN and SYK, are already known drug targets for RA. Of the remaining, 12 have direct PPI links to some of the current drug targets of RA. The scientific literature also provides evidences for the prominence of some of these 24 molecules in the pathogenesis of RA. These molecules, seemingly important to the cytokine signaling network, need to be further studied in order to establish their involvement in the pathogenesis of RA and to explore their potential for developing new therapeutics.

## Supporting information

S1 File The list of experimental methods used for determining protein-protein interactions in the six databases. (DOCX)

S2 File The human PPI database created from the six publicly available protein-protein interaction databases namely HPRD, BioGRID, IntAct, MINT, STRING and CRG. (TXT)

S3 File The list of search terms used in PubMed for retrieving articles which provided information on active cytokines and their target transcription factors. (DOC)

S4 File The intermediate proteins of the shortest paths. The number of occurrences of each of the intermediates is also mentioned in the file.
(TXT)

S5 File The edge list of the CPPIN network. (TXT)

S6 File An interaction map for 12 cytokines, eight transcription factors and 24 key molecules. (XGMML)

S1 Table In addition to 24, 10 other genes showed up in microarray data analysis after removing RA samples clustered with healthy controls. (XLSX)

S1 Fig Hierarchical clustering of RA and control samples based on the gene expression of selected key molecules in GSE12021 (HGU133B) after removing the RA sample clustered with the healthy controls. (TIF)

S2 Fig Hierarchical clustering of RA and control samples based on the gene expression of selected key molecules in GSE12021 (HGU133A) after removing the RA samples clustered with the healthy controls. (TIF)

S3 Fig Hierarchical clustering of RA and control samples based on the gene expression of selected key molecules in GSE55457 after removing the RA samples clustered with the healthy controls. (TIF)
