# Supplementary material for: A cytokine protein-protein interaction network for identifying key molecules in rheumatoid arthritis
Source: PLoS One. 2018 Jun 21;13(6):e0199530. doi: 10.1371/journal.pone.0199530 (PMC6013252; doi:10.1371/journal.pone.0199530)
Supplement: S1 File — (DOCX) [file pone.0199530.s001.docx]

A list of experimental methods that were used for determining protein-protein interactions in the six databases

BioGrid

| **S.NO.** | **Interaction detection method** |
| --- | --- |
| 1 | two-hybrid (MI: 0018) |
| 2 | fluorescence resonance energy transfer (FRET) (MI: 0055) |
| 3 | far western blotting (MI: 0047) |
| 4 | pull down (MI: 0096) |
| 5 | affinity chromatography (MI: 0004) |
| 6 | biochemical (MI: 0401) |
| 7 | X-ray crystallography (MI: 0114) |
| 8 | enzymatic study (MI: 0415) |

IntAct

| **S.NO.** | **Interaction detection method** |
| --- | --- |
| 1 | two-hybrid (MI: 0018) |
| 2 | FRET (MI: 0055) |
| 3 | far western blotting (MI: 0047) |
| 4 | pull down (MI: 0096) |
| 5 | Biochemical (MI: 0401) |
| 6 | X-ray crystallography (MI: 0114) |
| 7 | phage display (MI: 0084) |
| 8 | peptide array (MI: 0081) |
| 9 | co-immunoprecipitation (MI: 0019) |
| 10 | cross-linking study (MI: 0030) |
| 11 | beta lactamase complementation (MI: 0011) |
| 12 | filter binding (MI: 0049) |
| 13 | anti bait co-immunoprecipitation (MI: 0006 ) |
| 14 | two hybrid fragment pooling approach (MI: 0399) |
| 15 | surface plasmon resonance (MI: 0107) |
| 16 | dihydrofolate reductase reconstruction (MI: 0111) |
| 17 | beta galactosidase complementation (MI: 0010) |
| 18 | two hybrid pooling approach (MI: 0398) |
| 19 | interaction detection method (MI: 0001) |
| 20 | mammalian protein protein interaction trap (MI: 0231) |
| 21 | tandem affinity purification (MI: 0676) |
| 22 | anti tag co-immunoprecipitation (MI: 0007) |
| 23 | Enzyme-linked immunosorbent assay (ELISA) (MI: 0411) |
| 24 | co-migration in gel electrophoresis (MI: 0807) |
| 25 | protein kinase assay (MI: 0424) |
| 26 | two hybrid prey pooling approach (MI: 1112) |
| 27 | experimental interaction detection (MI: 0045) |
| 28 | two hybrid array (MI: 0397) |
| 29 | nuclear magnetic resonance (MI: 0077) |
| 30 | isothermal titration calorimetry (MI: 0065) |
| 31 | protease assay (MI: 0435) |
| 32 | affinity technology (MI: 0400) |

MINT

| **S.NO.** | **Interaction detection method** |
| --- | --- |
| 1 | two-hybrid (MI: 0018) |
| 2 | fluorescence resonance energy transfer (FRET) (MI: 0055) |
| 3 | far western blotting (MI: 0047) |
| 4 | pull down (MI: 0096) |
| 5 | affinity chromatography (MI: 0004) |
| 6 | biochemical (MI: 0401) |
| 7 | x-ray crystallography (MI: 0114) |
| 8 | phage display (MI: 0084) |
| 9 | peptide array (MI: 0081) |
| 10 | co-immunoprecipitation (MI: 0019) |
| 11 | cross-linking study (MI: 0030) |
| 12 | beta lactamase complementation (MI: 0011) |
| 13 | filter binding (MI: 0049) |
| 14 | anti bait co-immunoprecipitation (MI: 0006 ) |
| 15 | surface plasmon resonance (MI: 0107) |
| 16 | beta galactosidase complementation (MI: 0010) |
| 17 | two hybrid pooling approach (MI: 0398) |
| 18 | tandem affinity purification (MI: 0676) |
| 19 | anti tag co-immunoprecipitation (MI: 0007) |
| 20 | ELISA (MI: 0411) |
| 21 | co-migration in gel electrophoresis (MI: 0807) |
| 22 | protein kinase assay (MI: 0424) |
| 23 | experimental interaction detection (MI: 0045) |
| 24 | two hybrid array (MI: 0397) |
| 25 | nuclear magnetic resonance (MI: 0077) |
| 26 | isothermal titration calorimetry (MI: 0065) |
| 27 | protease assay (MI: 0435) |
| 28 | affinity technology (MI: 0400) |
| 29 | competition binding (MI: 0405) |
| 30 | protein three binding (MI: 0437) |
| 31 | molecular sieving (MI: 0071) |
| 32 | enzymatic study (MI: 0415) |
| 33 | blue native page (MI: 0276) |
| 34 | co-migration in non denaturing gel electrophoresis (MI: 0404) |
| 35 | cosedimentation (MI: 0027) |
| 36 | saturation binding (MI: 0440) |
| 37 | ubiquitin reconstruction (MI: 0112) |
| 38 | cosedimentation through density gradient (MI: 0029) |
| 39 | fluorescence technology (MI: 0051) |
| 40 | cosedimentation in solution (MI: 0028) |
| 41 | lambda phage display (MI: 0066) |
| 42 | circular dichroism (MI: 0016) |
| 43 | bioluminescence resonance energy transfer (MI: 0012) |
| 44 | biomolecular fluorescence complementation (MI: 0809) |
| 45 | mass spectrometry studies of complexes (MI: 0069) |
| 46 | solid phase assay (MI: 0892) |
| 47 | light scattering (MI: 0067) |
| 48 | biophysical (MI: 0013) |
| 49 | protein array (MI: 0089) |
| 50 | fluorescence polarization spectroscopy (MI: 0053) |
| 51 | neutral component (MI: 0497) |
| 52 | bait (MI: 0496) |
| 53 | fluorescence-activated cell sorting (MI: 0054) |
| 54 | phosphatase assay (MI: 0434) |
| 55 | protein cross-linking with a bifunctional reagent (MI: 0031) |
| 56 | classical fluorescence spectroscopy (MI: 0017) |
| 57 | ion exchange chromatography (MI: 0226) |
| 58 | x-ray scattering (MI: 0826) |
| 59 | dynamic light scattering (MI: 0038) |
| 60 | kinase scintillation proximity assay (MI: 0425) |
| 61 | electrophoretic mobility supershift assay (MI: 0412) |
| 62 | lex-a dimerization assay (MI: 0369) |
| 63 | three hybrid (MI: 0588) |
| 64 | x-ray fiber diffraction (MI: 0825) |
| 65 | thermal shift binding (MI: 1235) |
| 66 | fluorescence correlation spectroscopy (MI: 0052) |
| 67 | chromatography technology (MI: 0091) |
| 68 | protein complementation assay (MI: 0090) |
| 69 | fluorescence recovery after photobleaching (MI: 1016) |
| 70 | zymography (MI: 0512) |

CRG

| **S.NO.** | **Interaction detection method** |
| --- | --- |
| 1 | two-hybrid (MI: 0018) |
| 2 | fluorescence resonance energy transfer (FRET) (MI: 0055) |
| 3 | far western blotting (MI: 0047) |
| 4 | pull down (MI: 0096) |
| 5 | affinity chromatography (MI: 0004) |
| 6 | x-ray crystallography (MI: 0114) |
| 7 | co-immunoprecipitation (MI: 0019) |
| 8 | cross-linking study (MI: 0030) |
| 9 | anti bait co-immunoprecipitation (MI: 0006 ) |
| 10 | surface plasmon resonance (MI: 0107) |
| 11 | two hybrid pooling approach (MI: 0398) |
| 12 | anti tag co-immunoprecipitation (MI: 0007) |
| 13 | ELISA (MI: 0411) |
| 14 | nuclear magnetic resonance (MI: 0077) |
| 15 | protein three binding (MI: 0437) |
| 16 | molecular sieving (MI: 0071) |
| 17 | blue native page (MI: 0276) |
| 18 | cosedimentation (MI: 0027) |
| 19 | cosedimentation through density gradient (MI: 0029) |
| 20 | fluorescence technology (MI: 0051) |
| 21 | cosedimentation in solution (MI: 0028) |
| 22 | mass spectrometry studies of complexes (MI: 0069) |
| 23 | light scattering (MI: 0067) |
| 24 | protein cross-linking with a bifunctional reagent (MI: 0031) |
| 25 | classical fluorescence spectroscopy (MI: 0017) |
| 26 | ion exchange chromatography (MI: 0226) |
| 27 | chromatography technology (MI: 0091) |
| 28 | literature |
| 29 | co-affinity purification (COAP assay) |
| 30 | in vitro |
| 31 | in vivo |
| 32 | Physical interaction |
| 33 | immunoprecipitation |
| 34 | mass spectrometry |
| 35 | reaction |
| 36 | direct complex |
| 37 | chromatin immunoprecipitation assays (MI: 0402) |
| 38 | electrophoretic mobility shift assay (MI: 0413) |
| 39 | predictive text mining (MI: 0087) |
| 40 | x-ray diffraction |
| 41 | tandem affinity purification (TAP) |
| 42 | inferred by author (MI: 0363) |
| 43 | transcriptional complementation assay (0232) |
| 44 | confocal microscopy (MI: 0663) |
| 45 | electron microscopy (MI: 0040) |
| 46 | scintillation proximity assay (MI: 0099) |
| 47 | nucleic acid uv cross-linking assay (MI: 0430) |
| 48 | fluorescence microscopy (MI: 0416) |
| 49 | Affinity capture mass spectroscopy |
| 50 | imaging techniques |
| 51 | mass spectrometry |

HPRD

| **S.NO.** | **Interaction detection method** |
| --- | --- |
| 1 | yeast two-hybrid |
| 2 | in vitro |
| 3 | in vivo |

STRING

| **S.NO.** | **Interaction detection method** |
| --- | --- |
| 1 | experimental interaction detection (MI: 0045) |
