# Supplementary material for: A cytokine protein-protein interaction network for identifying key molecules in rheumatoid arthritis
Source: PLoS One. 2018 Jun 21;13(6):e0199530. doi: 10.1371/journal.pone.0199530 (PMC6013252; doi:10.1371/journal.pone.0199530)
Supplement: S3 File — (DOC) [file pone.0199530.s003.doc]

The list of terms used in the PubMed searches to retrieve articles which provided information on active cytokines and their target transcription factors

1. fibroblast-like synoviocytes[Title] OR FLS [Title] OR synovial fibroblasts[Title]) AND (rheumatoid[Title] OR arthritis[Title]) AND (TNF[Title] OR TNFα[Title] OR tumor necrosis factor[Title]) AND (signal[Title] OR signaling[Title] OR transduction[Title])

The retrieved references from this search are listed in the main manuscript: they are 9 (Lee A et al.), 56 (Akhtar N et al.), 57 (Trenkmann M et al.) and 58 (Xu H et al.).

1. (fibroblast-like synoviocytes[Title] OR FLS [Title] OR synovial fibroblasts[Title]) OR (rheumatoid[Title] OR arthritis[Title]) AND (TNF[Title] OR TNFα[Title] OR tumor necrosis factor[Title]) AND (gene[Title] OR expression[Title])

The retrieved reference (10, Sohn et al.) from this search is listed in the main manuscript

1. (fibroblast-like synoviocytes[Title] OR FLS [Title] OR synovial fibroblasts[Title] OR rheumatoid synoviocytes[Title]) AND (rheumatoid[Title] OR arthritis[Title]) AND (TNF[Title] OR TNFα[Title] OR tumor necrosis factor alpha [Title] OR IL-1β[Title]) AND (cytokine[Title] OR cytokines[Title] OR proinflammatory[Title])

The retrieved references from this search are listed in the main manuscript: they are 11 (Granet C et al.) and 58 (Xu H et al.).

1. (fibroblast-like synoviocytes[Title] OR synoviocyte[Title] OR FLS [Title] OR synovial fibroblasts[Title]) AND (rheumatoid[Title] OR arthritis[Title]) AND (inflammatory cytokines[Title]) AND (activation[Title] OR expression[Title] OR regulate[Title])

The retrieved references from this search are listed in the main manuscript: they are 12 (Angiolilli C et al.), 32 (Cheon H et al.) and 59 (Ji JD et al.).

1. (fibroblast-like synoviocytes[Title] OR synoviocyte[Title] OR FLS [Title] OR synovial fibroblasts[Title] OR fibroblast[Title]) AND (rheumatoid[Title] OR arthritis[Title]) AND (activation[Title] OR expression[Title] OR regulate[Title] OR induce[Title] OR induces[Title]) AND (cytokine[Title] OR chemokine[Title])

The retrieved references from this search are listed in the main manuscript: they are 13 (Morel JCM et al.), 16 (Wong CK et al.), 35 (Xing R et al.), 60 (Xu J et al.), 61 (Ahn JK et al.), 62 (Filer A et al.), 71 (Ahmed S et al.) and 63 (Han CW et al.).

1. (fibroblast-like synoviocytes[Title] OR synoviocyte[Title] OR FLS [Title] OR synovial fibroblasts[Title] OR fibroblast[Title]) AND (rheumatoid[Title] OR arthritis[Title]) AND (cytokine[Title] OR chemokine[Title] OR IL-17[Title] OR IL17[Title])

The retrieved references from this search are listed in the main manuscript: they are 14 (Hwang SY et al.), 23 (Lee EJ et al.), 16 (Wong CK et al.), 13 (Morel JCM et al.), 35 (Xing R et al.), 60 (Xu J et al.), 61 (Ahn JK et al.), 62 (Filer A et al.), 71 (Ahmed S et al.), 63 (Han CW et al.), 24 (Lee SY et al.), 64 (Tsuchiya A et al.), 69 (Kim HR et al.) and 73 (Kontny E et al.).

1. (fibroblast-like synoviocytes[Title] OR synoviocyte[Title] OR FLS [Title] OR synovial fibroblasts[Title] OR fibroblast[Title]) AND (rheumatoid[Title] OR arthritis[Title]) AND (cytokine[Title] OR IL-17[Title] OR interleukin-17[Title])

The retrieved references from this search are listed in the main manuscript: they are 23 (Lee EJ et al.), 31 (Kim KW et al.), 24 (Lee SY et al.), 16 (Wong CK et al.), 14 (Hwang SY et al.), 60 (Xu J et al.), 61 (Ahn JK et al.), 62 (Filer A et al.), 63 (Han CW et al.), 64 (Tsuchiya A et al.), 73 (Kontny E et al.) and 82 (Xing R et al.).

1. (rheumatoid[Title] OR arthritis[Title]) AND (NF-kappaB[Title] OR NFkb[Title] OR NF-kB[Title]) AND (signaling[Title])

The retrieved references from this search are listed in the main manuscript: they are 17 (Okamoto H et al.) and 57 (Trenkmann M et al.).

1. (rheumatoid[Title] OR arthritis[Title]) AND (fibroblast-like synoviocytes[Title] OR FLS[Title] OR synovial fibroblasts[Title]) AND (nuclear factor-kB[Title] OR NF-kappaB[Title] OR NFkb[Title] OR NF-kB[Title] OR pathway[Title]) AND (expression[Title] OR induced[Title] OR induce[Title] OR induces[Title] OR active[Title]) AND (interleukin[Title] OR IL-1β[Title]) AND (human[Title])

The retrieved references from this search are listed in the main manuscript: they are 18 (Liu FL et al.) and 74 (Yang CM et al.).

1. (rheumatoid[Title] OR arthritis[Title]) AND (fibroblast-like synoviocytes[Title] OR FLS[Title] OR synovial fibroblasts[Title]) AND (cells[Title])

The retrieved references from this search are listed in the main manuscript: they are 19 (Bartok B et al.), 79 (Zhu J et al.) and 80 (Ren J et al.).

1. (rheumatoid[Title] OR arthritis[Title]) AND (fibroblast-like synoviocytes[Title] OR FLS[Title] OR synovial fibroblasts[Title] OR synoviocytes[Title]) AND (TGF-beta[Title]) AND (pathway[Title] OR regulation[Title] OR active[Title] OR activated[Title] OR kinase[Title])

The retrieved references from this search are listed in the main manuscript: they are 20 (Hammaker DR et al.) and 33 (Sakuma M et al.).

1. (rheumatoid[Title] OR arthritis[Title]) AND (fibroblast-like synoviocytes[Title] OR FLS[Title] OR synovial fibroblasts[Title] OR synoviocytes[Title]) AND (pathway[Title] OR regulation[Title] OR active[Title] OR activated[Title] OR induce[Title] OR induces[Title] OR regulated[Title]) AND (light[Title])

The retrieved references from this search are listed in the main manuscript: they are 21 (Ishida S et al.), 22 (Kang YM et al.) and 75 (Shibata Y et al.).

1. (rheumatoid[Title] OR arthritis[Title]) AND (fibroblast-like synoviocytes[Title] OR FLS[Title]) AND (inflammatory[Title])

The retrieved references from this search are listed in the main manuscript: they are 26 (Angiolilli C et al.), 16 (Wong CK et al.), 65 (Kong QZ et al.), 66 (Lee H et al.), 67 (Igarashi H et al.), 68 (Lee YR et al.) and 76 (Bang JS et al.).

1. (rheumatoid[Title] OR arthritis[Title]) AND (STAT1[Title])

The retrieved reference (27, Kasperkovitz PV et al.) from this search is listed in the main manuscript.

1. (synoviocyte[Title] OR synoviocytes[Title]) AND (IFN regulatory factor 3[Title] OR IRF3[Title])

The retrieved reference (28, Sweeney SE et al.) from this search is listed in the main manuscript.

1. (synoviocyte[Title] OR synoviocytes[Title] OR synovial fibroblasts[Title]) AND (rheumatoid arthritis[Title]) AND (IL-22[Title] OR IL-22/IL-22R1[Title] OR interleukin-22[Title])

The retrieved references from this search are listed in the main manuscript: they are 15 (Kim KW et al.), 30 (Zhao M et al.), 29 (Carrión M et al.), 79 (Zhu J et al.) and 80 (Ren J et al.).

1. (synoviocyte[Title] OR synoviocytes[Title] OR synovial fibroblasts[Title]) AND (rheumatoid [Title] OR arthritis[Title]) AND (TGF-beta[Title] OR transforming growth factor[Title])

The retrieved references from this search are listed in the main manuscript: they are 33 (Sakuma M et al.), 20 (Hammaker DR et al.) and 81 (Warstat K et al.).

1. (synoviocyte[Title] OR synoviocytes[Title] OR synovial fibroblasts[Title]) AND (rheumatoid [Title] OR arthritis[Title]) AND (patients[Title]) AND (receptor[Title] OR activation[Title])

The retrieved references from this search are listed in the main manuscript: they are 34 (Charbonneau M et al.) and 77 (Lazzerini PE et al.).
