# Supplementary figures and images for: A cytokine protein-protein interaction network for identifying key molecules in rheumatoid arthritis
Source: PLoS One. 2018 Jun 21;13(6):e0199530. doi: 10.1371/journal.pone.0199530 (PMC6013252; doi:10.1371/journal.pone.0199530)

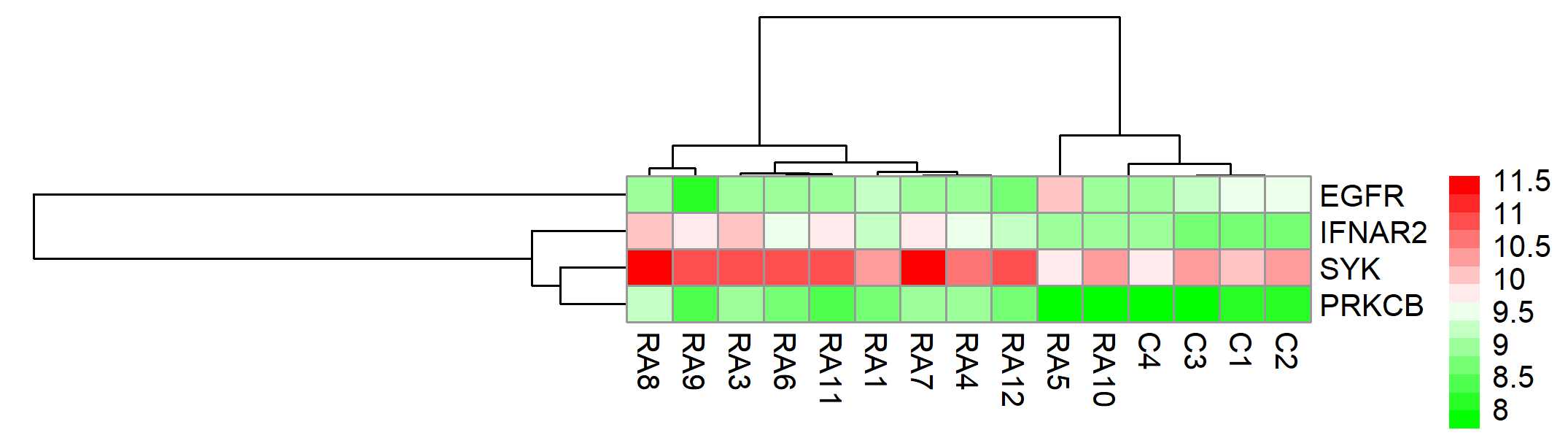

Supplement: S1 Fig — (TIF) [file pone.0199530.s008.tif]

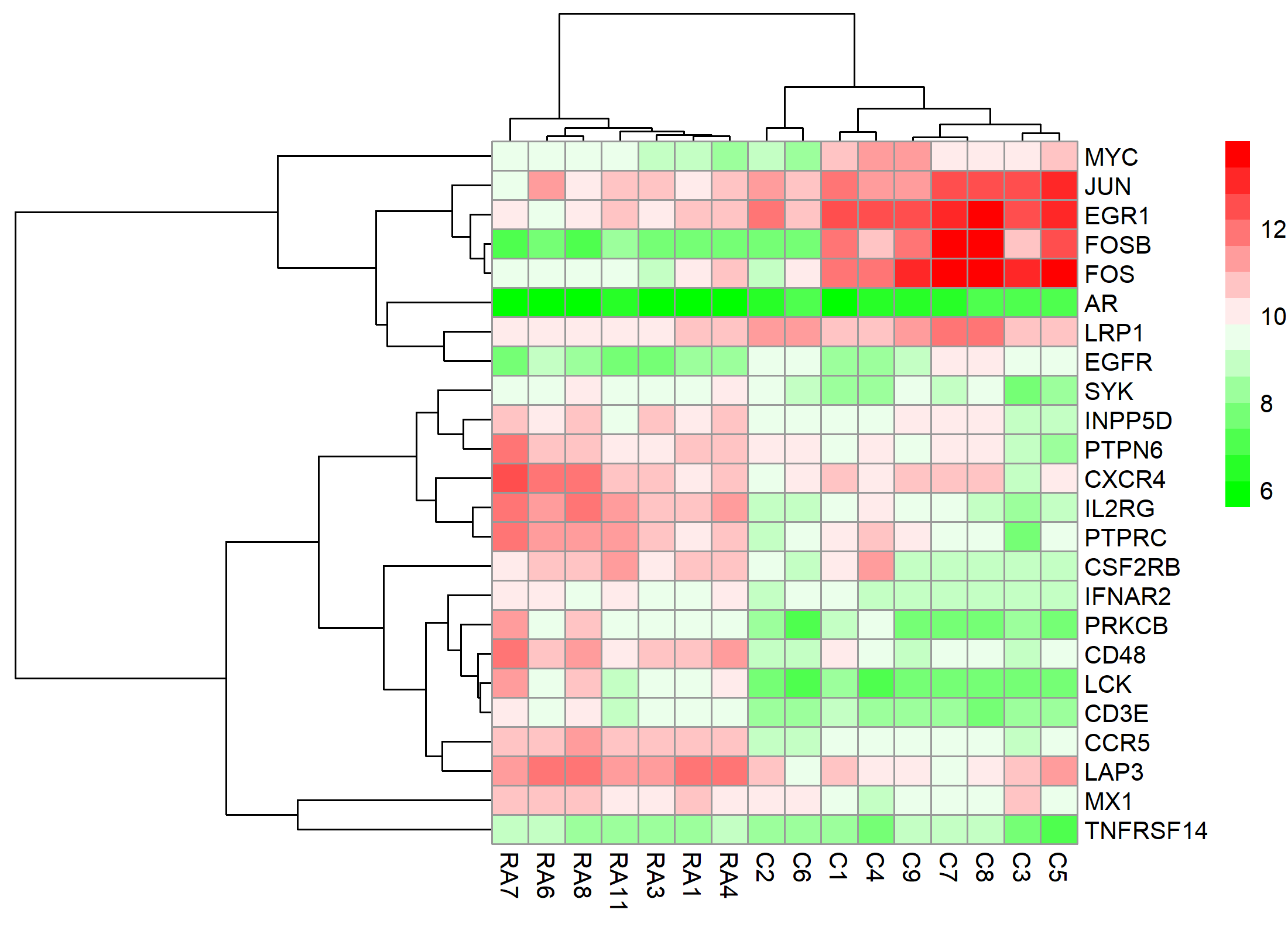

Supplement: S2 Fig — (TIF) [file pone.0199530.s009.tif]

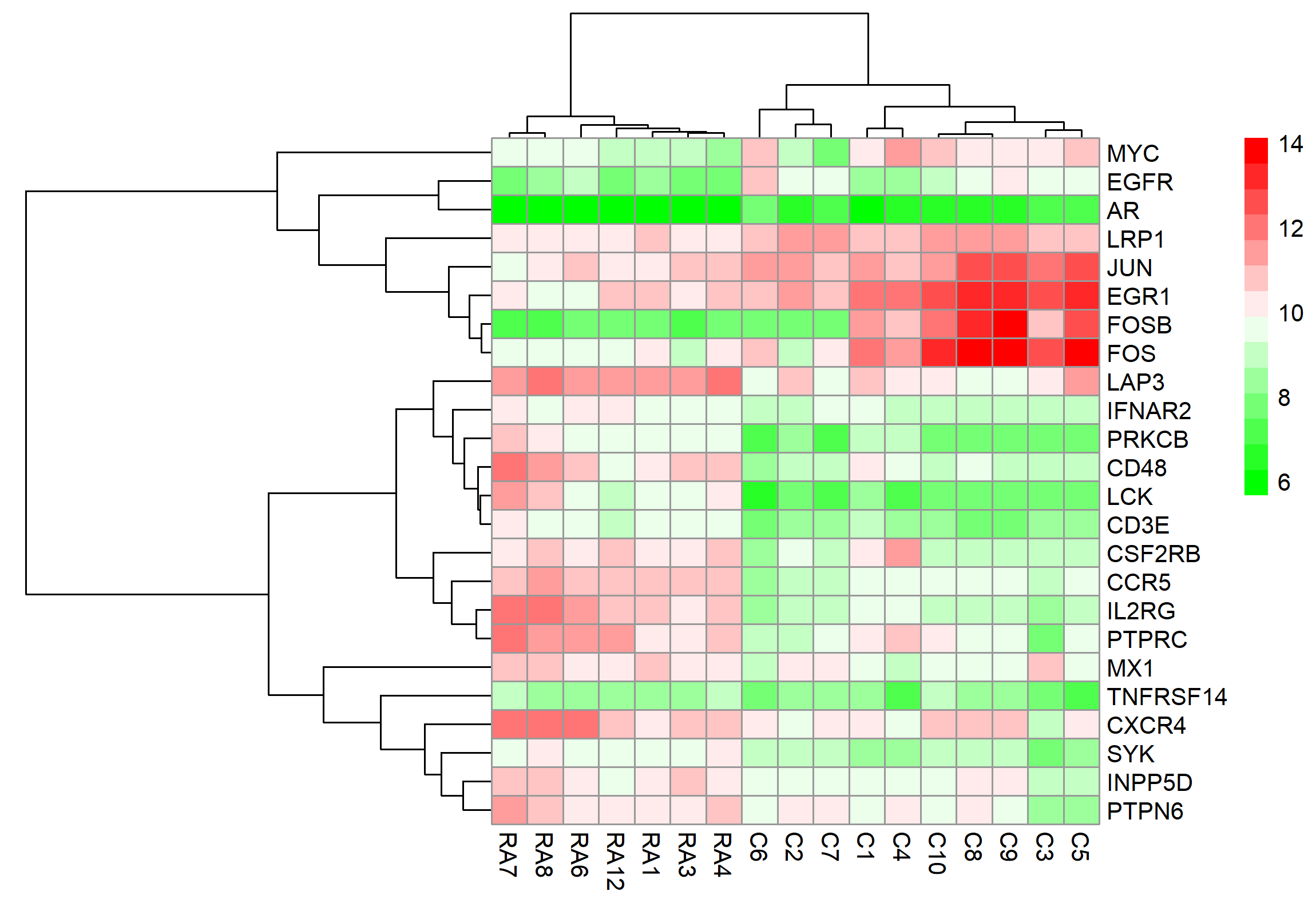

Supplement: S3 Fig — (TIF) [file pone.0199530.s010.tif]
